# Silicon nitride: a potent solid-state bioceramic **inactivator of ssRNA viruses**

**DOI:** 10.1038/s41598-021-82608-3

**Published:** 2021-02-03

**Authors:** Giuseppe Pezzotti, Francesco Boschetto, Eriko Ohgitani, Yuki Fujita, Wenliang Zhu, Elia Marin, Bryan J. McEntire, B. Sonny Bal, Osam Mazda

**Affiliations:** 1 Ceramic Physics Laboratory, Kyoto Institute of Technology, Sakyo-ku, Matsugasaki, Kyoto, 606–8585 Japan; 2 Department of Orthopedic Surgery, Tokyo Medical University, 6–7-1 Nishi-Shinjuku, Shinjuku-ku, Tokyo 160–0023 Japan; 3 The Center for Advanced Medical Engineering and Informatics, Osaka University, 2–2 Yamadaoka, Suita, Osaka 565–0854 Japan; 4 Department of Immunology, Graduate School of Medical Science, Kyoto Prefectural University of Medicine, Kamigyo-ku, 465 Kajii-cho, Kyoto, 602–8566 Japan; 5 Department of Dental Medicine, Graduate School of Medical Science, Kyoto Prefectural University of Medicine, Kamigyo-ku, Kyoto, 602–8566 Japan; 6 SINTX Technologies Corporation, 1885 West 2100 South, Salt Lake City, UT 84119 USA

**Keywords:** Biomaterials, Biomedical materials, Implants

## Abstract

Surface inactivation of human microbial pathogens has a long history. The Smith Papyrus (2600 ~ 2200 B.C.) described the use of copper surfaces to sterilize chest wounds and drinking water. Brass and bronze on doorknobs can discourage microbial spread in hospitals, and metal-base surface coatings are used in hygiene-sensitive environments, both as inactivators and modulators of cellular immunity. A limitation of these approaches is that the reactive oxygen radicals (ROS) generated at metal surfaces also damage human cells by oxidizing their proteins and lipids. Silicon nitride (Si_3_N_4_) is a non-oxide ceramic compound with known surface bacterial resistance. We show here that off-stoichiometric reactions at Si_3_N_4_ surfaces are also capable of inactivating different types of single-stranded RNA (ssRNA) viruses independent of whether their structure presents an envelop or not. The antiviral property of Si_3_N_4_ derives from a hydrolysis reaction at its surface and the subsequent formation of reactive nitrogen species (RNS) in doses that could be metabolized by mammalian cells but are lethal to pathogens. Real-time reverse transcription (RT)-polymerase chain reaction (PCR) tests of viral RNA and in situ Raman spectroscopy suggested that the products of Si_3_N_4_ hydrolysis directly react with viral proteins and RNA. Si_3_N_4_ may have a role in controlling human epidemics related to ssRNA mutant viruses.

## Introduction

ssRNA viruses possess a very simple structure consisting of a protein-shell capsid surrounding their genetic material. They are classified into enveloped and non-enveloped types depending on the presence or not in their structure of a protective outermost layer mainly composed of phospholipids and proteins derived from the host cells, while also including viral glycoproteins that serve as binders to receptor sites on the membrane of the host cell. An additional classification stands from the RNA viruses containing single-stranded RNA genomes of positive or negative polarity, which are referred to as positive-stranded RNA (+ ssRNA) and negative-stranded RNA (-ssRNA) viruses, respectively.

The significantly higher mutation rates of ssRNA viruses as compared to DNA viruses can lead to an increased probability of disease transmission because their diversity makes it difficult to produce effective vaccines to prevent it. High mutation rates in a number of ssRNA viruses are a consequence of RNA polymerases lacking proofreading ability^1–3^. Although, viral infections can be controlled through vaccination and antiviral drugs, the high mutation rates of ssRNA virions requires annual updates, a strategy limited by the limited number of approved vaccine approaches and antiviral drugs, as well as by their side effects. For example, M2 ion channel blockers and neuraminidase inhibitors are used to treat influenza; however, both approaches are limited by the capacity of viruses to promptly induce viral genetic changes in their genome^4^.

Environmental viral disinfection strategies are either based on chemical actions (e.g., formaldehide, acetylethylenimine, etc.) or physical agents such as ultraviolet light, ion, electron, or gamma irradiations^5^. Chemical disinfectants can be toxic to cells because they react rapidly with free thiols^6,7^ and amine groups^8^ in proteins and DNA. Exposure to exogenous formaldehide plays a role in age-related impairment and dementia^9^. Radiations-based disinfection has its drawbacks too, since even small doses of radiation can cause DNA and protein damage and alterations in cell growth^10^. Moreover, both chemical and physical disinfecting methods result in partial viral inactivation only^11^. New antiviral strategies are therefore desirable, targeting a broad spectrum of viral infections, including new outbreaks, while maintaining safety toward mammalian cells.

We describe here the possibility of a rapid ssRNA viral inactivation mediated by simple contact with the surface of non-oxide Si_3_N_4_ ceramic, independent of the virus being of an enveloped or non-enveloped type, or possessing a RNA genome with positive or negative polarity. We hypothesize that the antiviral mechanisms induced at the Si_3_N_4_ surface are multifactorial, being based on electrical charge attraction (including competitive binding to an envelope glycoprotein hemagglutinin in the case of influenza virus) and viral RNA cleavage by ammonia and other RNS (common to all types of virus tested). These synergic mechanisms both rely on the peculiar surface chemistry of Si_3_N_4_ in aqueous environment, which elutes ammonium/ammonia with a slow and controlled kinetics, while spontaneously developing free electrons and negatively charged silanol surface sites in aqueous solution^12–14^. Micrometric Si_3_N_4_ powder was tested against three different types of ssRNA viruses at different temperatures in aqueous environment. The surface chemistry of the Si_3_N_4_ powder was also altered by thermal treatments in different pH environments, in order to understand the roles of differently charged surface sites involved with viral inactivation. This study represents an experimental confirmation with different ssRNA viruses of a recent report showing almost instantaneous “catch-and-kill” inactivation of the SARS-CoV-2 virus by the same Si_3_N_4_ particles used in the present experiments^15^. Safe to the human health and to the environment, Si_3_N_4_ offers a unique chance in achieving almost instantaneous surface inactivation of microbial pathogens without the toxicity associated with other anti-viral strategies.

## Materials and methods

### Characterizations of the antiviral Si_*3*_*N*_*4*_* powder*

Bulk Si_3_N_4_ samples (manufactured by SINTX Corporation; Salt Lake City, UT) were sintered in nitrogen atmosphere at a temperature in excess of 1700 °C, and densified by hot isostatic pressing under a N_2_ gas pressures > 200 MPa and at temperature exceeding 1650 °C. The produced Si_3_N_4_ mainly consisted of anisotropic β-Si_3_N_4_ grains separated by thin (< 2 nm) grain boundaries of amorphous or crystalline yttrium aluminum oxynitride or Si(Y)AlON, respectively. The Si_3_N_4_ powder used in this antiviral study was obtained from the above sintered samples by mechanical grinding the Si_3_N_4_ sintered body and through successive filtration. This grinding/filtration process allowed us to statistically control the particle size within a desired interval.

A photoelectron spectrometer (JPS-9010 MC; JEOL Ltd., Tokyo, Japan) with an X-ray source of monochromatic MgK (output 10 kV, 10 mA) was employed for X-ray photoelectron spectroscopy (XPS) analyses. Prior to characterization, the surface was cleaned by Ar^+^ sputtering in the pre-chamber, while actual measurements were conducted in the vacuum chamber at around 2 × 10^–7^ Pa with an analyzer pass energy of 10 eV and voltage step size of 0.1 eV. X-ray incidence and takeoff angles were set at 34° and 90°, respectively. Spectra were averaged over ten separate measurements.

Cathodoluminescence (CL) spectra were collected using a field-emission gun scanning electron microscope (FEG-SEM, SE-4300, Hitachi Co., Tokyo, Japan) equipped with an optical device. The acceleration voltage and the beam current were fixed at 5 kV and 80 pA, respectively. The CL device consisted of an ellipsoidal mirror and an optical fiber bundle, which served to collect and to address the emitted electron-stimulated luminescence into a highly spectrally resolved monochromator (Triax 320, Jobin–Yvon/Horiba Group, Tokyo, Japan). About 100 CL spectra were randomly obtained from different areas using an acquisition time of 60 s in order to ensure statistical significance. The spectra, which were characteristic of quite shallow portions of material (~ 5 nm in depth), were deconvoluted using commercial software (Origin 9.1, OriginLab Co., Northampton, MA, USA) and the results averaged and compared.

Attenuated total reflection Fourier transform infrared (ATR-FTIR) spectra were also recorded by means of a high sensitivity spectroscope (Spectrum 100FT-IR Spotlight 400; PerkinElmer Inc., Waltham, MA, USA). The spectral resolution of this equipment was 0.4 cm^−1^. Average ATR-FTIR spectra were computed using 6 independent measurements performed on n = 3 samples. Spectral acquisition and pre-processing of raw data, which included baseline subtraction, smoothing, normalization and fitting, were carried out using commercially available software (Origin 8.5, OriginLab Co., Northampton, MA, USA).

### Cells, viruses, and immunochemistry characterizations

MDCK cells (Madin-Darby canine kidney cell) were purchased from DS Pharma Biomedical Co., Ltd. (Suita, Japan). The MDCK cells were cultured in DMEM (Nacalai Tesque, Kyoto, Japan) supplemented with 4% FBS, 100 U/ml penicillin, and 100 μg/ml streptomycin (Complete Medium) and plated in 6-well plate at 6 × 10^5^ cells/well for plaque assay, or loaded on glass based dishes (TECHNO GLASS Co., Shizuoka, Japan) at 1 × 10^6^ cells/dish for immunochemistry assays. Cells were cultured in Complete Medium at 37 °C in an atmosphere containing 5% CO_2_.

The CRFK (feline kidney) cell line was purchased from Health Science Research Resources Bank (Sennan, Japan), while the Rhesus monkey kidney (LLC-MK2) cell line was purchased from ATCC. Both types of cell were cultured in DMEM supplemented with 10% FBS, 100 U/ml penicillin, and 100 μg/ml streptomycin and plated in 96-well plate at 3 × 10^4^ cells/well for TCID_50_ assay.

The influenza A virus, A/Puerto Rico/8/34(H1N1) (PR8) strain, was obtained from the Virus Research Center, National Institute of Infectious Diseases. The HEV71 (ATCC VR­1432) and the FCV F-9 strain (ATCC VR-782) were directly purchased from ATCC. A brief description of the viral strains used in this study is given in the supplementary materials.

The Si_3_N_4_ powder was added to the virus solution to a concentration of 15 wt.% or 30 wt.%, followed by mixing for 1, 5, 10, and 30 min at room temperature (RT) or 4 °C using a rotating equipment. After centrifugation at 12,000 rpm for 2 min at 4 °C, the viral infectivity of the supernatant was compared with that of sham exposure by plaque assay (Influenza A H1N1) or TCID50 assay (FCV and HEV71). A schematic draft of the inoculation and subsequent testing/characterization procedures is offered in the Supplementary Information.

For the plaque assay, a confluent monolayer of MDCK cells in a 6-well plate was washed twice with serum-free DMEM (SF DMEM), followed by infection with 100 μl of virus suspension in a tenfold serial dilution. After incubation at 37 °C for 1 h with tilting every 10 min, unabsorbed inoculum was removed, and infected cells were overlaid with 4 ml of DMEM containing 2.5 μg/ml trypsin (Sigma-Aldrich Co. LLC, Saint Louis, USA) and 0.2% albumin (Wako Pure Chemical Industries, Ltd., Osaka, Japan). The plate was incubated at 37 °C in an atmosphere of 5% CO_2_ for 2 days. For plaque counting, cells were fixed with 5% glutaraldehyde solution for 2 h, the agarose medium was removed, and the cells were stained with 1% crystal violet.

After removal of the culture supernatant, a confluent monolayer of CRFK or LLC-MK2 cells in a 96-well plate was inoculated with 50 μl of virus suspension in a tenfold serial dilution. After incubation at 37 °C for 1 h with tilting every 10 min, unabsorbed inoculum was removed, and infected cells were added with 100 μl of DMEM containing 4% FBS, 0.2% albumin and 2.5 μg/ml of trypsin. The plate was incubated at 37 °C in an atmosphere of 5% CO_2_ for 6–8 days. Cytopathic effect (CPE) was observed in all wells, and TCID_50_ calculated by the Reed-Muench method. Statistical analyses of the results were carried out by means of the unpaired Student’s *t*-test. The statistical significance of the data was evaluated as highly statistically relevant (*p* < 0.01; labeled with two asterisks), statistically relevant (*p* < 0.01; labeled with one asterisk), or non-significant (*n.s.*).

In order to confirm the infectivity of viruses exposed or unexposed to Si_3_N_4_ particles, we visualized MDCK cells inoculated with Influenza A virions exposed and non-exposed to Si_3_N_4_ powder in the fluorescence microscope after washing the infected cells with TBS (20 mM Tris–HCl pH 7.5, 150 mM NaCl), fixing them with 4% paraformaldehyde for 10 min at RT, and permeabilizing them with 0.1% triton X in TBS for 5 min at RT. Successively, the cells were blocked with 2% skim milk in TBS for 60 min at RT, and stained with mouse anti-influenza A virus nucleoprotein antibody (red) (AA5H ab20343, Abcam, Cambridge, UK) (Dilution = 1:500) and fluorescence signals from cell F-actin (green) (Thermo Fisher Scientific) (Dilution = 1:500) for 60 min at RT. After washing with a washing buffer, cells were incubated with an Alexa 549 Goat Anti-mouse IgG F(ab')2 (Thermo Fisher Scientific, MA, USA) (Dilution = 1:250) for 60 min at RT in the dark. Three samples each were prepared for Si_3_N_4_ exposed and non-exposed tests, respectively. Three visual fields were observed of each sample under a fluorescence microscope (BZX710; Keyence, Osaka, Japan), subsequently the total number of cells and the number of infected cells were calculated using Keyence BZ-X Analyzer. Statistical analyses of the results were carried out by means of the unpaired Student’s *t*-test (*p* < 0.001; labeled with three asterisks).

The RNA of viruses exposed or unexposed to Si_3_N_4_ particles was extracted using a QI Amp Viral RNA Mini Kit (QIAGEN N.V., Hilden, Germany) according to the manufacturer protocol*.* Reverse transcription was performed using a ReverTra Ace qPCR RT Master Mix (TOYOBO CO., LTD**.**, Osaka, Japan). 8 μl of RNA template was added to 2 μl of 5 X RT Master Mix. The mixture was incubated at 37 °C for 15 min, 50 °C for 5 min, 98 °C for 5 min and 4 °C hold.

We selected primers and probes for Influenza A H1N1 virus based on genomic regions highly conserved in various subtypes and genotypes of the Influenza virus A (matrix protein gene). The fluorogenic probe for the Influenza A virus consisted of oligonucleotides with the 5′ reporter dye 6-carboxyfluorescein (FAM) and the 3′ quencher dye 6-carboxytetramethylrhodamine (TAMRA). A 20-μl PCR was performed using 2 μl of cDNA, 10 μl of KAPA PROBE FAST qPCR Master Mix (KAPA BIOSYSTEMS, Massachusetts, USA) containing ROX, 900 nM each influenza virus A primer, and 100 nM each probe. Amplification and detection were performed with an ABI StepOnePlus Real Time PCR System (under the following conditions: 1 cycle of 20 s at 95 °C, 45 cycles of 1 s at 95 °C and 20 s at 60 °C. During amplification, the System detector monitored real-time PCR amplification by quantitatively analyzing fluorescence emissions. The reporter dye (FAM) signal was measured against the internal reference dye (ROX) signal to normalize for non-PCR-related fluorescence fluctuations occurring from well to well. The threshold cycle represented the refraction cycle number at which a positive amplification reaction was measured and was set at 10 times the standard deviation of the mean baseline emission calculated for PCR cycles 3 to 15.

In situ Raman spectra were collected using a highly sensitive instrument (LabRAM HR800, Horiba/Jobin–Yvon, Kyoto, Japan) with a 20 × optical lens. The spectroscope operated in microscopic measurement mode with confocal imaging in two dimensions. A holographic notch filter within the optical circuit was used to efficiently achieve high-resolution spectral acquisitions. A spectral resolution of 1.5 cm^−1^ was obtained using a 532 nm excitation source operating at 10 mW. The Raman emission was monitored by means of a single monochromator connected to an air-cooled charge-coupled device (CCD) detector (Andor DV420-OE322; 1024 × 256 pixel). The acquisition time was fixed at 10 s. Thirty spectra were collected and averaged at each analysis time-point. Raman spectra were deconvoluted into Gaussian–Lorentzian sub-bands using commercially available software (LabSpec 4.02, Horiba/Jobin–Yvon, Kyoto, Japan).

## Investigated ssRNA viruses

Three ssRNA viruses were investigated, which were purposely selected with different structural and isoelectric point (IEP) characteristics, as follows: Influenza A/Puerto Rico/8/1934 H1N1 (-ssRNA; enveloped; IEP = 7.5; referred to as Influenza A H1N1, henceforth), Feline calicivirus (FCV; + ssRNA; non enveloped; IEP = 3.9), and Enterovirus 71 (EV-A71; + ssRNA; non enveloped; IEP = 10.9). These three viral entities were selected because of their different genomic and virion structures, surface charge, and because of their propensity to drift. The individual molecular composition of the capsid, envelope proteins, and RNA of the selected viruses conferred on them distinct specificity and infectivity characteristics. More detailed descriptions of the used viruses with related references are given in the Supplementary Information.

## Si_3_N_4_ powder and its surface reactions in aqueous solution

**Figure 1:**
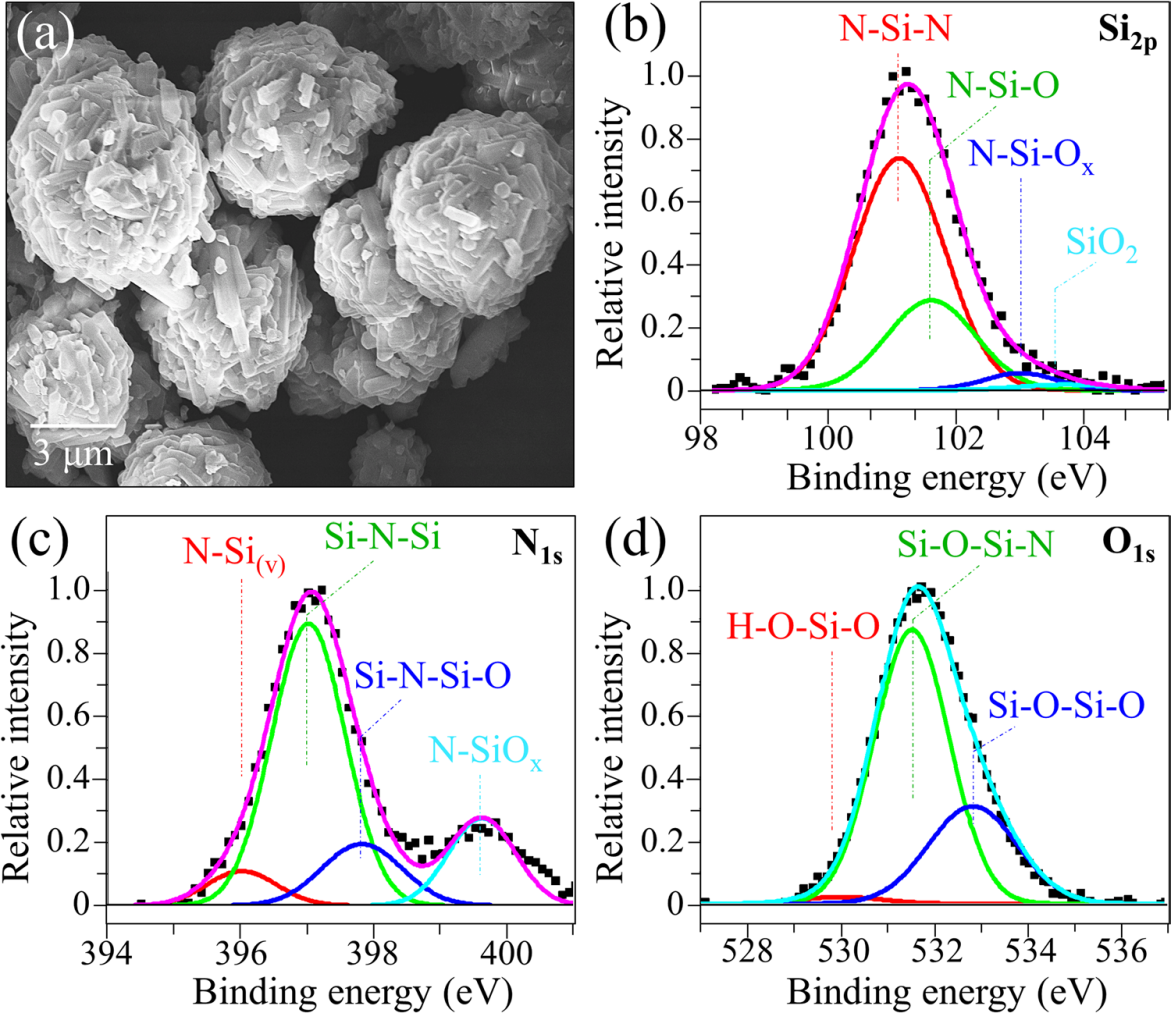
Characterizations of the as-received Si_3_N_4_ powder: (**a**) scanning electron micrograph reveals size and morphology of the powder. In (**b**)–(**d**), XPS narrow scans at the Si_2p_, N_1s_, and O_1s_ edges with related deconvolution in sub-bands representing different bond populations at the surface of the material (cf. labels).

A scanning electron microscopy (SEM) image of the Si_3_N_4_ powder is shown in Fig. 1a. The observation revealed a relatively homogeneous and roundish morphology of the Si_3_N_4_ powder with an average diameter of 6 μm and a standard deviation of ± 1.6 μm. The micrometric Si_3_N_4_ particles were in turn composed of smaller elongated crystallites, which imparted to the particles a relatively rough surface in the sub-micrometric scale, thus increasing the specific surface area that potentially interacts with the virions. X-ray photoelectron spectroscopy (XPS) characterizations of the Si_3_N_4_ powder are shown in Fig. 1b–d for Si_2p_, N_1s_, and O_1s_, respectively. The morphology of the Si_2p_ edge, shown in Fig. 1b, revealed four sub-bands deconvoluted from the overall spectrum. From low toward high binding energies, those sub-bands can be assigned to N-Si–N, N-Si–O, N-Si-O_x_, and O-Si–O bonds (cf. labels). The N_1s_ and O_1s_ edges basically confirmed the presence of these surface bond population (Fig. 1c, d, respectively), but the former edge also revealed a minor fraction of N sites bonded to Si vacancies (cf. N-Si_(v)_ sub-band in Fig. 1c). The set of XPS data shows that the solid surface of Si_3_N_4_ terminates with amphoteric silanols (Si–OH) and basic secondary amine (Si_2_-NH) groups. An additional set of surface chemistry and stoichiometry characterizations of the micrometer sized Si_3_N_4_ powder used in this study is given in the Supplementary Information (Fig. S-1).

Data collected by attenuated total reflection Fourier transform infrared (ATR-FTIR) and cathodoluminescence (CL) spectroscopies basically confirmed the XPS data in showing that the surface of Si_3_N_4_ is a zwitterionic one, being mainly composed of amphoteric silanols and basic silylamine surface groups with minor fractions of nitrogen vacancy sites, *V*_*N*_^3+^, Si dangling bonds (N_3_Si), N dangling bonds (Si_2_N), and N_4_^+^ defects associated with the presence of N–N bonds^16^.

In aqueous environment dissociation/protonation reactions take place, according to which dissociation of amine surface sites and the subsequent formation of Si–OH bonds result in the separation of ammonium ions (NH_4_^+^) or ammonia (NH_3_) and in the creation of additional surface silanols^17^. According to ab initio calculations^18,19^, the most energetically favorable path for Si_3_N_4_ hydrolysis can be represented, as follows:(1)1

According to Eq. (1), the nitrogen ions that elute from the Si_3_N_4_ surface predominantly become NH_4_^+^ and NH_3_ at low and high (> 9.25) pH values, respectively. At physiological pH, the release of nitrogen species leaves positively charged N-vacancies at the surface, which partly counterbalance negatively charged deprotonated silanols (Si–O^−^). It also generates free electrons, which in turn induce splitting of the surrounding water molecules and contribute to create transient ROS and, consequently, a variety of RNS including hydroxylamine, NH_2_OH, and, ultimately, nitric oxide, NO^12^.

A consequence of these transient off-stoichiometric reactions is a robust pH buffering, which stabilizes the local pH in the neighborhood of the Si_3_N_4_ surface at around 8.5^12–14^. A direct confirmation of ammonium/ammonia elution has recently been recorded as a function of pH using colorimetric ammonia assays after placing bulk Si_3_N_4_ samples into vials containing water^14^. Silicon elution was also found according to inductively coupled plasma atomic emission spectroscopy. Conversely, the elution of Si ions took several orders of magnitude longer time than that of N. Despite the balance alteration of charged surface groups, silanol solubility proceeds at a relatively slow rate at physiological pH and could be instantaneously promoted only under extremely alkaline pH values (i.e., > 10)^20–22^.

In Si_3_N_4_, the relative proportion of each surface group depends on both extent of surface hydrolysis and environmental pH. However, secondary silylamine are generally preponderant among basic sites because they are by far more resistant to hydrolysis than the primary silylamine sites^23,24^. In the present context, the N lone pair at the (stable) secondary silylamine sites adsorbed on the surface of Si_3_N_4_ is key in the electrostatic interaction between ssRNA viruses and Si_3_N_4_ particles, as explained later.

**Figure 2:**
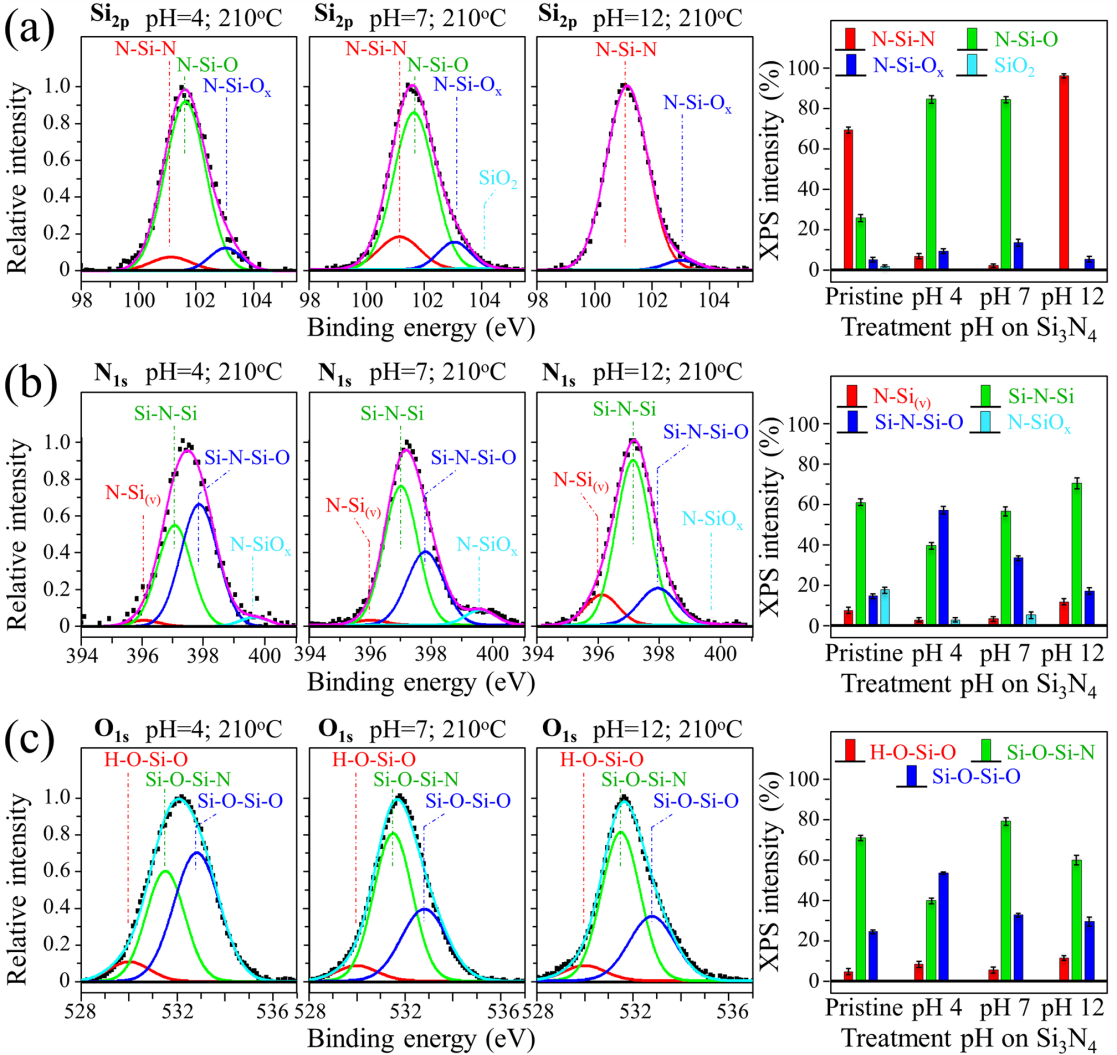
Deconvoluted XPS spectra of Si_3_N_4_ after 48 h autoclave treatment at 210 °C in aqueous environment with pH values of 4, 7, and 12 (cf. labels in inset); narrow scans of Si_2p_, N_1s_, and O_1s_ edges are given in (**a**)–(**c**), respectively. The fractions of different surface bond populations (cf. labels in inset) are given in graphs on the right side of each plot.

In an attempt to delve into the electrostatic interaction between Si_3_N_4_ powder and virions, we attempted to alter the surface bond population of the Si_3_N_4_ particles upon pH treatments in acidic and alkaline environments (210 °C; 48 h under pH = 4, 7, and 12). Figure 2a–c show the Si_2p_, N_1s_, and O_1s_ edges of XPS narrow scans conducted on the Si_3_N_4_ powder as a function of treatment pH. ATR-FTIR spectra of the Si_3_N_4_ powder treated in strongly acidic and alkaline aqueous environment are shown in Figs. S-2(a) and (b), respectively, of the Supplementary Information. These sets of XPS and ATR-FTIR analytical data revealed that the balance between silanol and amine bond populations on the particle surface could indeed be altered to some extent in both acidic and alkaline environments. The results of narrow scan at the Si_2p_ edge (in Fig. 2a) show that it is possible to maximize the population of N-Si–N bonds at the Si_3_N_4_ surface at the expenses of the N-Si–O ones upon treating the powder in a strongly alkaline environment. This trend is confirmed by the narrow scan at the N_1s_ and O_1s_ edges (in Fig. 2b, c, respectively). On the other hand, the acidic environment was more effective in increasing the population of N-Si–O above N-Si–N bonds than the alkaline one^23^. However, the latter treatment also introduced new types of bonds, which included Si–OH and H–N–H^24^, as shown by the ATR-FTIR data in Fig. S-2(b).

**Figure 3:**
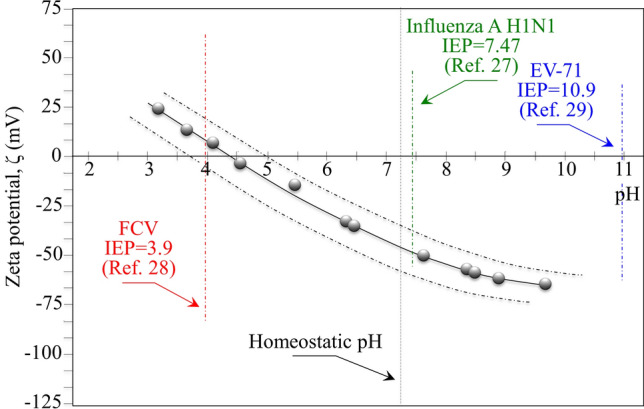
Results of zeta potential, ζ, measurements on the Si_3_N_4_ powder and its IEP value compared to the IEPs of the investigated viral strains as obtained from literature (cf. labels in inset).

It should be noted that, despite the observed variations in surface bond balance, silanol terminals and secondary amines were always the two dominating adsorbed species. This is best seen in the XPS O_1s_ narrow scans in Fig. 2c). These results are consistent with previous studies^25,26^ that identified pH < 4 as a threshold value for neutral Si–OH species to become the only surface terminating species. A direct consequence of bond balance alterations is the variation in surface IEP (Fig. 3). The variations in IEP values as a function of treatment in aqueous environment at different pH fluctuated in the range + 0.5/− 0.8 around the value 4.5 recorded for the as-received powder (cf. broken curves in Fig. 3). As a consequence, the net surface charge of the Si_3_N_4_ particles remained largely negative relative to homeostatic pH, with only a minor fraction of positively charged sites stemming from both SiNH_3_^+^ species and 3 + charged N-vacancies. Figure 3 also shows the IEPs of the viruses investigated in this study, as reported in literature^27–29^.

Previous investigations on bulk Si_3_N_4_ samples showed the possibility of modulating charging and wetting properties of the surface^30^. In principle, alterations of the surface bond structure of Si_3_N_4_ could be obtained through the concurrent application of temperature and pH environments. However, substantial variations toward a lower IEP value could only occur upon exposing the Si_3_N_4_ surface to temperatures > 1000 °C in oxygen atmosphere^30^. In this section, we confirmed that both silanols and secondary silylamine surface groups contribute to Si_3_N_4_ surface charging in aqueous medium. At homeostatic (or higher) pH, the former species deprotonates and thus charge negative, while the latter species possess an N lone pair capable of additional carbon bonding. While the balance between silanol and silylamine groups can be altered by pH treatments, the two species always coexist and intermix on the Si_3_N_4_ surface. As a result, one would expect that the electrostatic interaction between viruses and Si_3_N_4_ surface is only partially affected by the development of acidic and alkaline biological environments.

## Immunochemistry characterizations

### Influenza A H1N1 virions

The effect of the Si_3_N_4_ surface on H1N1 infection was analyzed in Madin-Darby Canine Kidney (MDCK) cell culture. MDCK cells are widely used to examine infectivity of Influenza virus, because they are highly susceptible to this virus. Their infection showed replication kinetics similar to that obtained upon infecting human cells^31^. The MDCK cell line is also a suitable choice for efficiently supporting replication and, consequently, for correctly addressing the receptor binding preference of various viruses^32–34^.

The virus was preliminary exposed in solution to a 15 wt.% fraction of Si_3_N_4_ powder in solution for different times (between 1 and 30 min; referred to as “virus inactivation times”, henceforth) prior to infecting the MDCK cells. Si_3_N_4_ particles were removed through filtration and centrifugation, and the MDCK cells were inoculated with the virus samples for 48 h before infectivity evaluation. Viral infectivity was compared to that of unexposed virus samples tested under the same conditions as negative controls (cf. the complete description in the Supplementary Information and a schematic draft of the testing procedure in Fig. S-3). The plaque assay was applied as a standard method in determining virus concentration in terms of infectious dose.

**Figure 4:**
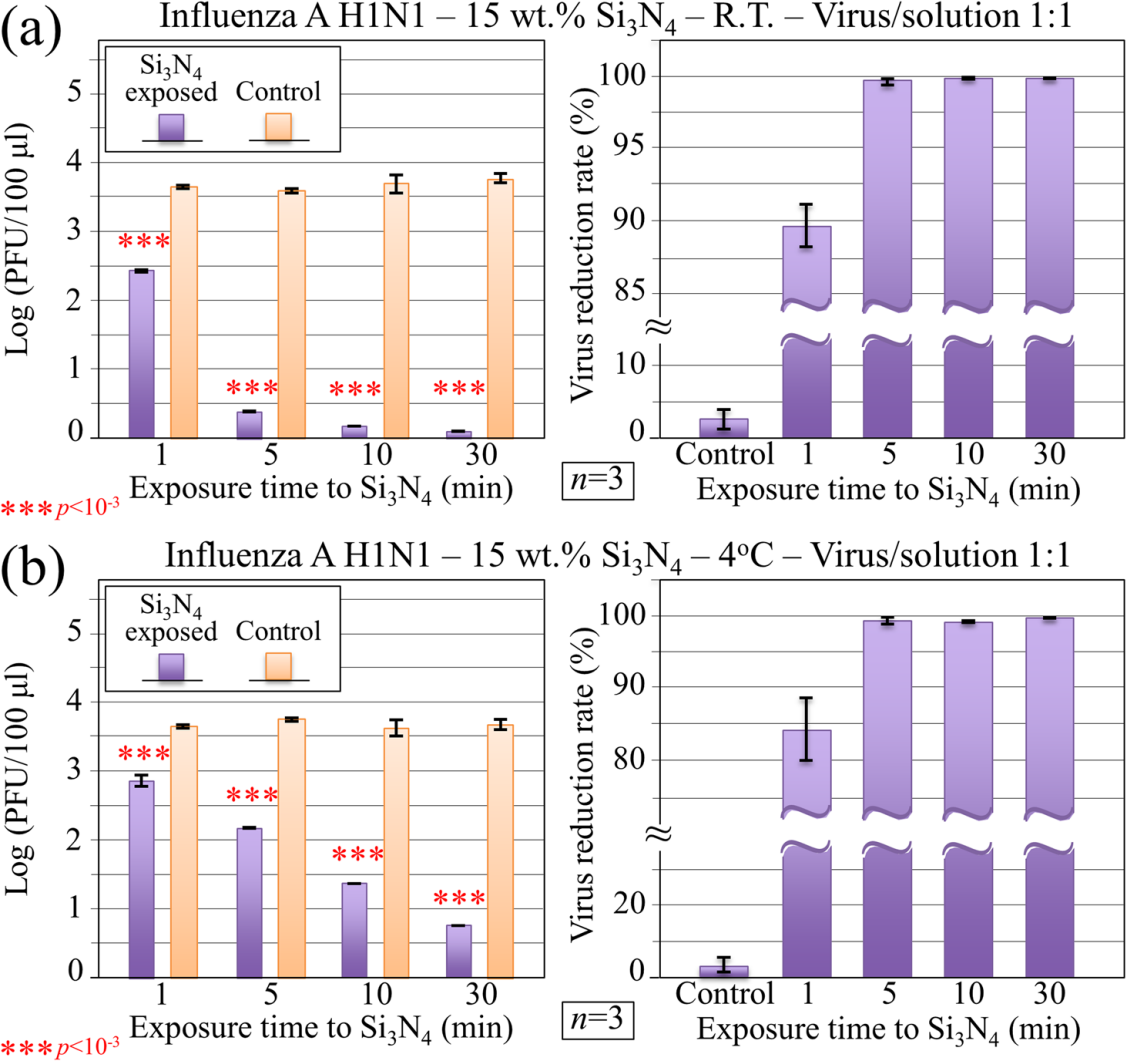
Immunochemistry results collected on MDCK cells infected with Influenza A H1N1 virus at room temperature (R.T.) and at 4 °C in (**a**) and (**b**), respectively. Data are plotted (with statistical validation) in logarithmic scale as PFU counts and virus reduction rate as a function of virus exposure time to 15 wt.% Si_3_N_4_ in aqueous solution. Control samples refer to virions embedded for the same time in aqueous environment in absence of the Si_3_N_4_ powder.

We counted the number of plaques in cell samples and assessed virus quantities as plaque forming units PFU/100 μL as a function of virus inactivation time. Figure 4a, b show the results (with their related statistical validation) of these experiments (*n* = 3) plotted in logarithmic scale for inoculation experiments conducted at room temperature and 4 °C, respectively. Comparisons are made with control cell samples inoculated with virions unexposed to Si_3_N_4_ (labeled as “control”). The respective plots of reduction rate of infectious virus are also shown in the above figures. At room temperature (Fig. 4a), the PFU counts decreased and the reduction rate increased sharply as compared to control with increasing virus inactivation times. Remarkably, the reduction rate already reached ~ 90% (*p* > 0.001 in Student’s test; *n* = 3) after exposing the virus for only 1 min to the Si_3_N_4_ powder. For exposure times equal to or longer than 10 min reduction rates > 99.8% were recorded for all cell samples inoculated with Si_3_N_4_-exposed virions. A reduction of temperature to 4 °C apparently increased the viral load since a slower decrease rate (~ 84%) was recorded for 1 min exposure to Si_3_N_4_. However, with increasing Si_3_N_4_ exposure time to 5 min or longer the reduction rate was always > 99% (Fig. 4b). This set of experiments consistently clarified that the Si_3_N_4_ powder substantially inactivated the H1N1 Influenza A virus within exposure times as short as few minutes.

**Figure 5:**
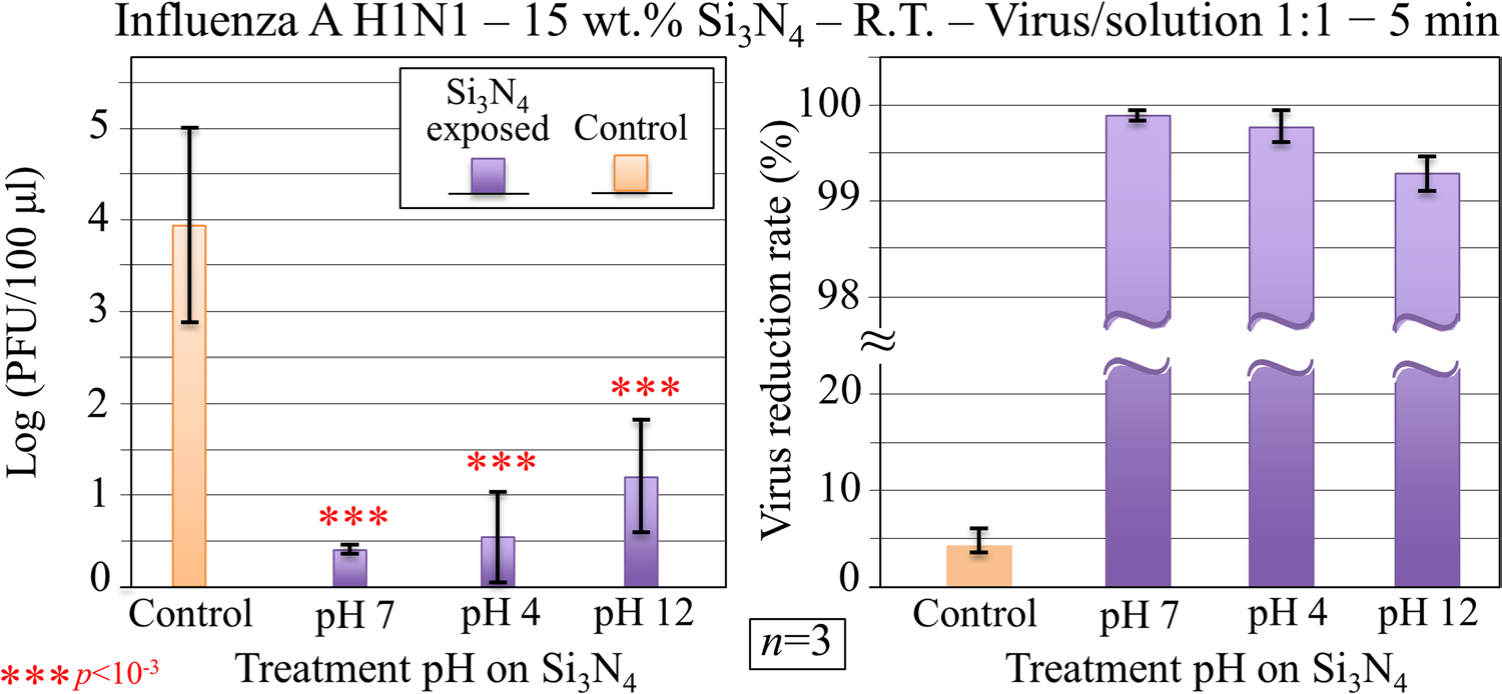
Immunochemistry results collected on MDCK cells infected with Influenza A H1N1 virus exposed for 5 min to aqueous solution containing 15 wt.% Si_3_N_4_ powder at room temperature. Data are plotted (with statistical validation) in logarithmic scale as PFU counts and virus reduction rate as a function of the pH of a 48 h autoclave pre-treatment of Si_3_N_4_ powder at 210 °C. Control samples refer to virions embedded for 5 min in aqueous environment in absence of the Si_3_N_4_ powder.

Figure 5 shows the results of an additional set of experiments conducted at room temperature under the same conditions as above, but using Si_3_N_4_ powder with surface charge conditions altered by pH treatments in either acidic or alkaline environment (210 °C; 48 h under pH = 4, 7, and 12). Pre-treatment of virions with surface-charge-altered Si_3_N_4_ powders was conducted for 5 min. Data in Fig. 5 show a slightly lowered effectiveness for virions treated with Si_3_N_4_ powder exposed to a strongly alkaline environment, although a virus reduction rate > 99% could be reached independent of pH. As previously described in Sect. "Investigated ssRNA viruses", exposing the Si_3_N_4_ powder to a strongly alkaline environment enabled maximizing the surface population of N-Si–N bonds at the expenses of the N-Si–O, while the acidic environment was effective in increasing the population of N-Si–O above N-Si–N bonds. However, despite such an altered balance in surface molecular species, the surface of Si_3_N_4_ always preserved its zwitterionic nature including silanols and secondary silylamine groups in aqueous medium. Data in Fig. 5 suggest that a reduced population of surface silanols through heavy alkaline treatment only led to a slightly lowered antiviral effectiveness of the Si_3_N_4_ powder against Influenza A H1N1 virus.

### FCV and EV-A71 virions

**Figure 6:**
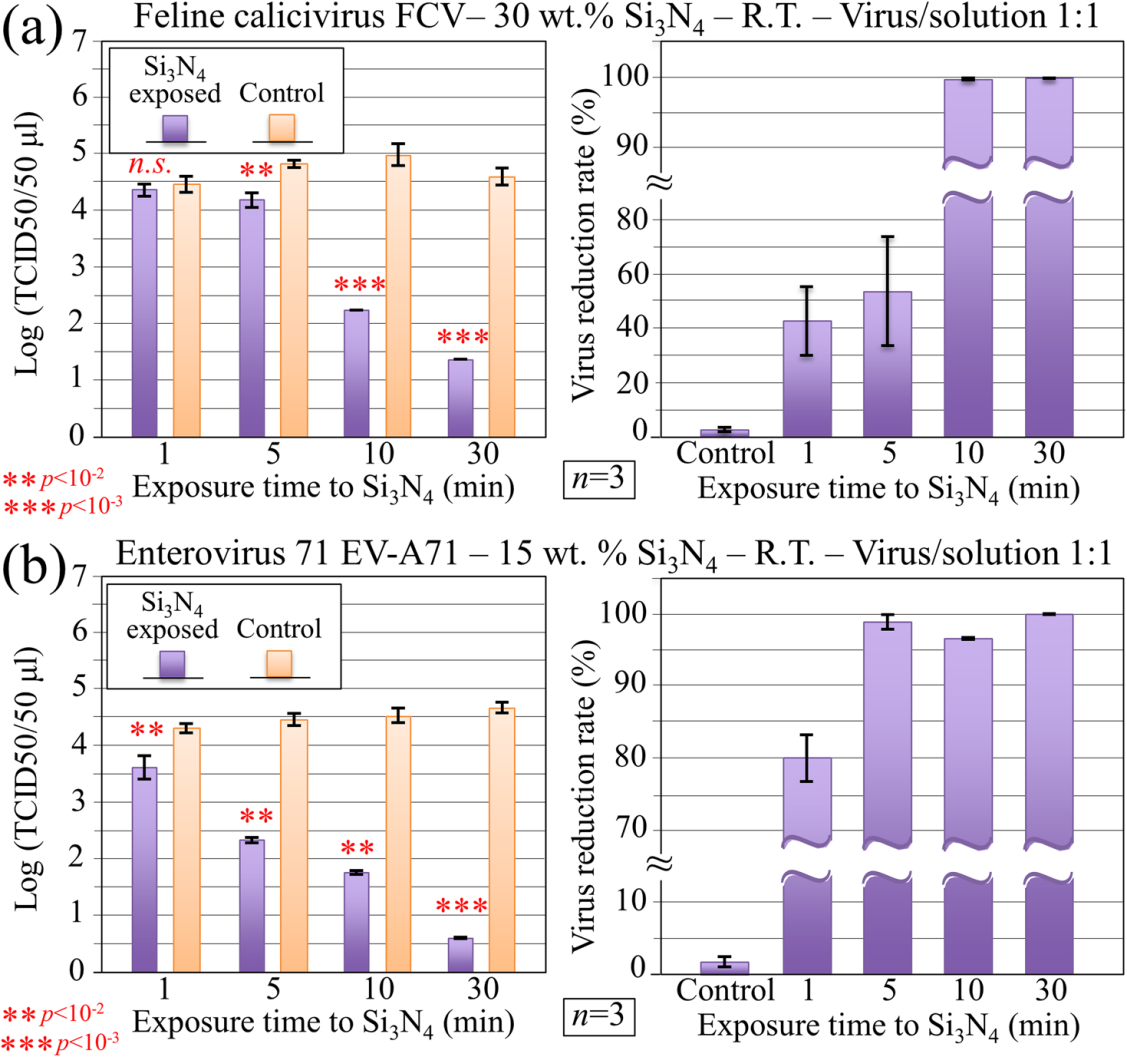
Immunochemistry results collected on: (**a**) CRFK cells infected with FCV virus; and, (**b**) LLC-MK2 cells infected with EV-A71 virus. In both cases, data were collected at room temperature (R.T.) and plotted (with statistical validation) in logarithmic scale as TCID counts and virus reduction rate as a function of virus exposure time to Si_3_N_4_. The fractions of Si_3_N_4_ powder contained in the aqueous solution used in virus pre-treatment are given in inset.

Experiments conducted with the same protocol as above were then applied to quantitatively assess virus inactivation efficiency upon pre-treating FCV and EV-A71 virions with the Si_3_N_4_ powder. The former virus was inoculated into cat kidney cortex epithelial cells (CRFK line). This line of feline kidney cells has extensively been used for viral infectivity assays and for studying the biology of various retroviruses and derived vectors^35^. The EV-A71 virus was tested against rhesus monkey kidney cells (LLC-MK2 line), because these cells were found persistently infected with simian virus and developed into a stable carrier state characterized by extensive viral proliferation without obvious cytopathic effects^36^. Figure 6a, b show the results (with their related statistical validation) of median tissue culture infectious dose (TCID_50_) and reduction rate of infectious virus as a function of virus inactivation time for the cases of FCV and EV-A71 inoculation in CRFK and LLC-MK2 cells, respectively. Data (*n* = 3) on CRFK and LLC-MK2 cells were collected at room temperature with inoculating virions previously treated in aqueous solutions containing 30 and 15 wt.% Si_3_N_4_, respectively. A comparison of the inactivation efficiency of Si_3_N_4_ toward different viruses (cf. Figs. 4a and 6) revealed that the FCV was the most resistant to inactivation: even with a 30% fraction of Si_3_N_4_ powder during pre-treatment of virions, double the time (10 vs. 5 min) was needed to achieve a nearly full inactivation as compared to H1N1 Influenza A virions. On the other hand, the inactivation trend for EV-A71 was very similar to that observed for the H1N1 Influenza A strain (15% Si_3_N_4_ powder in the inactivating solution in order to reach virus reduction rate > 99% with 5 min virion exposure to Si_3_N_4_ particles).

The above immunochemistry characterizations suggest that a chemical interaction occurs between ssRNA viruses and the Si_3_N_4_ surface in aqueous environment. Such a chemical interaction leads to a high degree of virus inactivation. Although the details of inactivation mechanisms might slight differ among the viral species studied, a substantial inactivation effect could commonly be achieved within minutes upon adjusting the fraction of Si_3_N_4_ particles in the inactivating medium. To this extent, we shall assume that the observed viral inactivation is commonly related to the phenomenon of Si_3_N_4_ hydrolysis in aqueous environment as represented by Eq. (1). The viral inactivation effect persisted after thermally treating the Si_3_N_4_ powder in different pH environments, although a relatively small decrease in antiviral efficiency could be found upon exposing the Si_3_N_4_ powder to a strongly alkaline environment (cf. Fig. 5). The key to interpret such differences likely relates to the electrical charge of surface groups and the IEP of the Si_3_N_4_ surface. This point will be discussed in a later section.

## Fluorescence imaging and RT-PCR analyses of gene fragmentation

**Figure 7:**
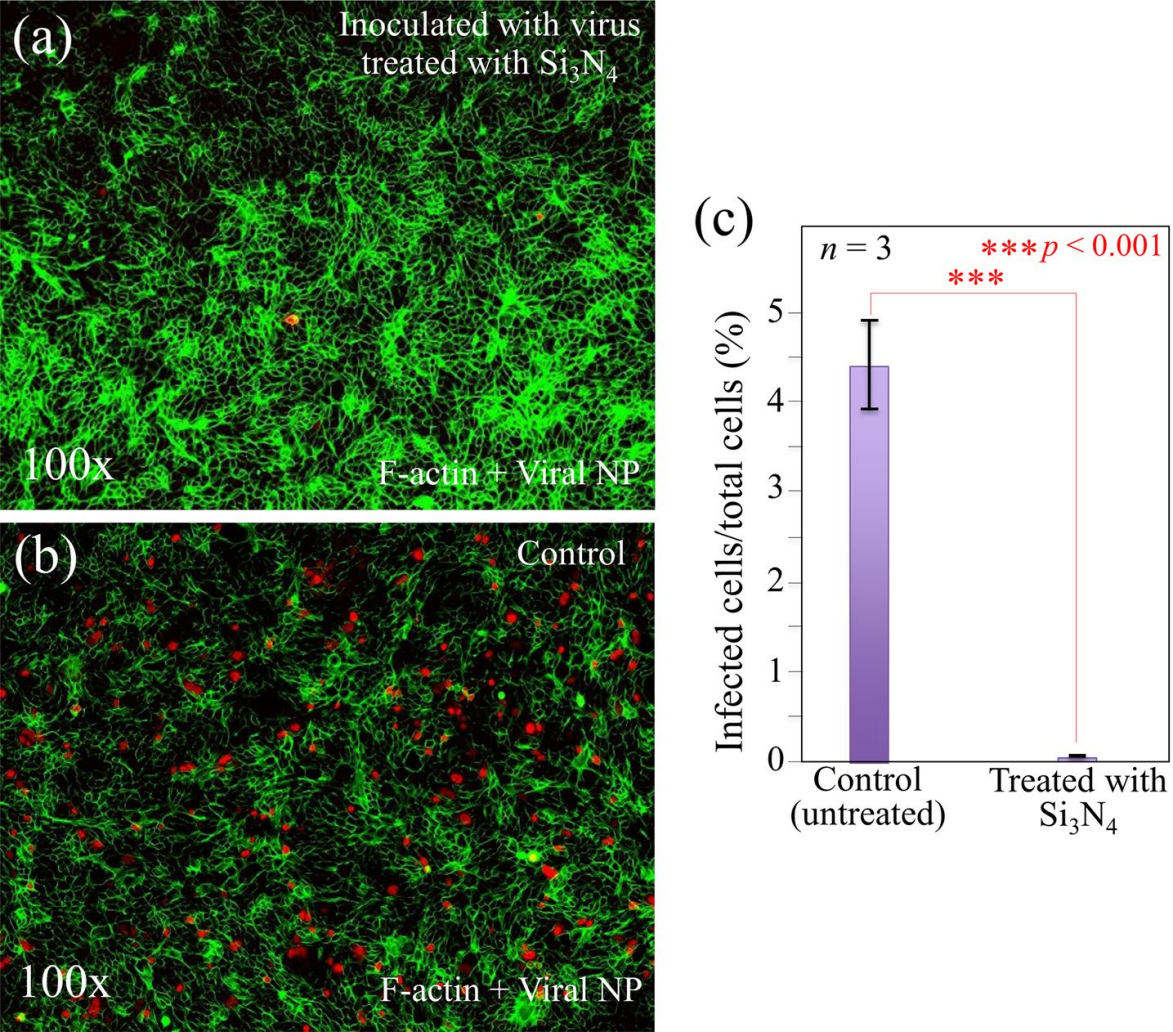
Fluorescence micrographs of the MDCK cell population inoculated with Influenza A H1N1 virions (green cell F-actin; red viral nucleoproteins): (**a**) cells inoculated with virions that were preliminarily exposed for 5 min to 15 wt.% Si_3_N_4_-containing aqueous solution; and, (**b**) cells inoculated with virus exposed for 5 min to pure aqueous solution (control sample). In (**c**), quantification with related statistical validation of infected cells is obtained upon direct counting on fluorescence micrographs. The full set of fluorescence micrographs for both samples is shown in Figs. S-4 and S-5 of the Supplementary Information.

In order to substantiate the virus inactivation effect as recorded by immunochemistry characterizations, we performed fluorescence assays on inoculated MDCK cells and TaqMan-based real-time reverse transcription (RT)-polymerase chain reaction (PCR) analyses^37^ on the Influenza A H1N1 viral strain before and after inactivation upon contact with Si_3_N_4_ powder. Figure 7 shows fluorescence micrographs of the MDCK cell population associated with viral RNA. Fluorescent staining was performed 9 h after infection. Cells inoculated with unexposed virus (control sample), but subjected to an otherwise identical procedure, and cells inoculated with Influenza A H1N1 virions, which were preliminarily exposed for 5 min to 15 wt.% Si_3_N_4_ in aqueous solution are imaged in (a) and (b), respectively. Both micrographs display the overlap of fluorescence signals from cell F-actin (green) and virus nucleoproteins (NP; red); the latter one being localized in the cells’ cytoplasm, while the former stemming in the cell surrounding. The complete set of fluorescence data (*n* = 3) are shown in the Supplementary Information (Figs. S-4and S-5 for the cases of inoculated virions being untreated and pre-treated with Si_3_N_4_, respectively). The comparison shows an almost complete lack of viral infection upon using virions exposed to the Si_3_N_4_ powder and provides a clear visual confirmation of the immunochemistry data in Fig. 4, thus visualizing the antiviral effect of Si_3_N_4_. A quantification based on counting virus nucleoproteins on fluorescence micrographs is offered in Fig. 7c.

In order to obtain information about the viral RNA structure after surface interactions with Si_3_N_4_ powder, we performed RT-PCR assay on the viral RNA of Influenza A H1N1 supernatant virions exposed and non-exposed to Si_3_N_4_ powder at room temperature. Two viral concentrations (i.e., 1.0 × 10^7^ and 5.0 × 10^5^ PFU/ml) were tested for a fixed 15 wt.% Si_3_N_4_ powder concentration in aqueous solution. Independent of viral concentration, the RT-PCR tests revealed the occurrence of significant RNA fragmentation for the three selected probes (cf. selected primers and probes for TaqMan amplification of cDNA with Influenza A H1N1 virus sequence and quantitative results of RT-PCR tests in Tables 1 and 2, respectively). We interpreted these results as an experimental evidence of RNA fragmentation by hydrolysis-driven reactions at the virion/Si_3_N_4_ interface.

**Table 1:** Selected primers and probes for TaqMan amplification of viral RNA from Influenza A H1N1 virus.

Target	Primer or probe	Sequence	Nucleotide positions
A (M gene)	INFA-1	5′ GGACTGCAGCGTAGACGCTT	217–236
A (M gene)	INFA-2	5′ CATCCTGTTGTATATGAGGCCCAT	382–405
A (M gene)	INFA-3	5′ CATTCTGTTGTATATGAGGCCCAT	277–300
A (M gene)	INFA probe	5′ CTCAGTTATTCTGCTGGTGCACTTGCCA	349–376

**Table 2:** Quantitative results (n = 3) of RT-PCR tests on viral RNA from Influenza A H1N1 virus unexposed (Control) and supernatant virions after exposure to Si3N4 powder (Supernatant). Two different viral concentrations were tested as indicated in the left column of the table. In n = 3 tests performed on Si3N4 powder pellets (not shown in the table), the viral RNA was undetermined.

Sample type	Sample number	C_T_	C_T_ mean
1.0 × 10^7^ PFU/ml	Control 1	26.02254	25.32722
	Control 2	25.1734	
	Control 3	24.78571	
	Supernatant 1	28.39764	29.04330
	Supernatant 2	28.80912	
	Supernatant 3	29.92314	
5.0 × 10^5^ PFU/ml	Control 1	29.90594	29.41437
	Control 2	28.68238	
	Control 3	29.65478	
	Supernatant 1	32.50948	33.64089
	Supernatant 2	33.37704	
	Supernatant 3	35.03614	

## Raman spectroscopy of Si_3_N_4_-exposed and unexposed virions

**Figure 8:**
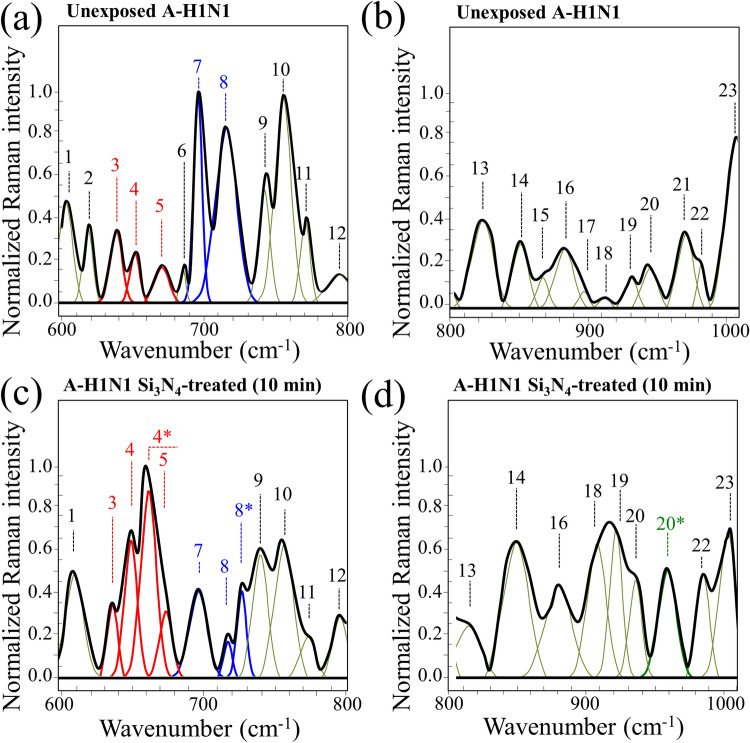
Deconvoluted Raman spectra in the frequency ranges 600–800 cm^−1^ (**a**) and 800–1000 cm^−1^ (**b**), as collected in situ on a sample of pristine Influenza A H1N1 virus; in (**c**) and (**d**), Raman spectra are given in the same frequency intervals, respectively, as collected on virions exposed for 10 min to a 15 wt.% Si_3_N_4_ dispersion in aqueous solution. The deconvoluted Raman bands, labeled from 1 to 24, are listed according to their frequency at maximum and classified with respect to their vibrational origins in Tables S-I and S-II of the Supplementary Information.

In situ Raman experiments were performed on Influenza A H1N1 virions before and after 10 min exposure to 15 wt.% Si_3_N_4_ powder in aqueous solution. Figure 8a shows a Raman spectrum in the frequency range 600 ~ 800 cm^−1^, which was collected in situ on the unexposed population of Influenza A-H1N1 virions (control sample). According to general classifications^38^, the examined Raman spectral region in this range mainly contained vibrational features from C-S bonds and from ring vibrations in specific RNA nucleotides. In this spectral zone, we detected 12 main Raman bands in the control sample of H1N1 virions (cf. Fig. 8a and Table S-I for vibrational frequencies, physical origins of the 12 labeled bands, and related references). In Fig. 8b, the Raman spectrum of the unexposed virion population is shown in the frequency interval 800 ~ 1010 cm^−1^. In this zone, additional 11 bands were detected and labeled with respect to their frequency, physical origin, and related references as given in Table S-II. The Raman signals in this zone mainly included S–H bending modes in addition to ring vibrations from nucleotides and O–P–O stretching vibrations in RNA backbone. In the present context, a relevant vibrational signal was represented by Band 18, which is mainly related to S–H in-plane bending of homocysteine^39^. The same vibrational mode also contributed Band 22, although in strong overlap with Amide III signals from proteins^40^.

Substantial morphological differences could be found by comparing spectra of the same viral strain before and after entering in contact with the Si_3_N_4_ powder. A more extensive description and discussion of the complete spectrum of H1N1 virions and MDCK cells have been given in a previously published paper including a Raman follow-up of the viral inoculation process^41^. In this paper, we only discuss two fundamental spectral features related to structural alterations of methionine and RNA in the H1N1 virions after entering in contact the with the Si_3_N_4_ particles. Bands 3, 4, 5, 7, and 8 (at 640, 650, 669, 698, and 716 cm^−1^, respectively) in the Raman spectrum of untreated virions (Fig. 8a) are related to C-S bond stretching in the methionine residue^39,40,42–46^, which is the first standard amino acid residue at the N-terminal position of hemagglutinin (HA). More specifically, Bands 3 and 4 represent C-S bond vibrations on the CH_2_ side for the molecule in the *gauche* configuration, while Band 5 arises from the same C-S bond on the CH_2_ side for the molecule in the *trans* configuration. Conversely, Bands 7 and 8 relate to C-S stretching on the CH_3_ carboxyl side in *gauche* and *trans* configurations, respectively^42^. Upon comparing the Raman spectra of H1N1 virions before and after 10 min. treatment with 15 wt.% Si_3_N_4_ in water solution (in Fig. 8a, c, respectively), the most striking differences were related to C-S bond vibrations and consisted in a significant intensity reduction of Bands 7 and 8, while a substantial enhancement could be found in the relative intensity of Band 4 and a less pronounced one for Band 5. Concurrently, two new bands appeared, which were shifted toward higher frequencies with respect to Bands 4 and 8 and were labeled as Bands 4* and 8*, respectively (cf. Fig. 8c). The origin of these two new bands can be related to modifications of the methionine structure. More precisely, Bands 4* and 8* could be attributed to C–C-S stretching on the CH_2_ side and C-S-C stretching on the CH_3_ side of methionine in thioether configuration^39^. This point will be discussed in more details later. However, based on these observations, we hypothesize that the several concomitant spectral modifications detected for C-S bond-related vibrations suggest that the methionine residue of HA promptly interacted with the Si_3_N_4_ surface. In turn, such interaction induced fundamental modifications of its thioether group and terminal structure.

Comparing higher frequency spectra in Fig. 8b, d, additional differences could be located between the unexposed (control) viruses and the Si_3_N_4_-treated ones, as follows:

(i) An increased signal for Bands 18, 19, and 22 (located at 913, 929, and 985 cm^−1^, respectively) for Si_3_N_4_-exposed virions (Fig. 8d). All these bands are contributed by signals from homocysteine: Bands 18 and 22 correspond to the in-plane S–H bending mode, while Band 19 arises from C–C stretching^39^. Note, however, that no “pure” signal for homocysteine S–H vibration is available, since Band 18 overlaps a ring deformation mode of RNA adenine^46^ and the stretching mode of C-COO^−^ deprotonated carboxyl group^43^, Band 19 is also contributed by RNA adenine signals^46^, and Band 22 includes Amide III signals from proteins^40^ (cf. Table S-II).
(ii) Bands 15, 17, and 21 (at 872, 889, and 970 cm^−1^, respectively), which represent deformation modes of the guanine ring^46^, completely disappeared.
(iii) A new Band 20* at around 959 cm^−1^ appears, which corresponds to S–H in-plane bending vibration of homocysteine^39^, but it is also contributed by O–P–O symmetric stretching of adenosine monophosphate^47^.

The meaning of the spectral modifications recorded after exposing the virions to Si_3_N_4_ powder is discussed in the remainder of this section based on basic Raman experiments on individual methionine polymorphs in aqueous solution after interactions with the Si_3_N_4_ powder.

**Figure 9:**
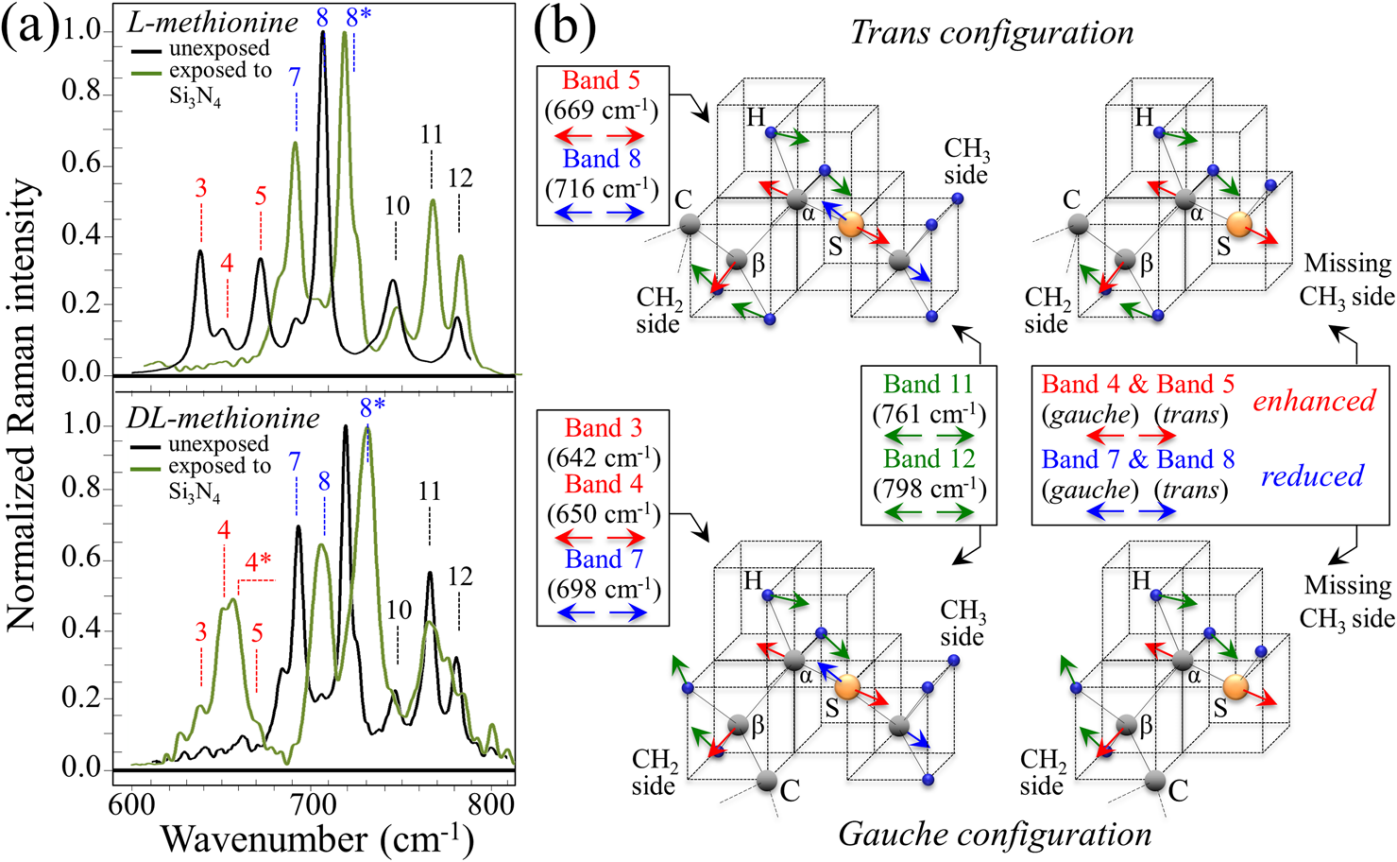
(**a**) Raman spectra of *L-* and *LD-*methionine polymorphs in aqueous solution with and without 15 wt.% Si_3_N_4_ dispersion; spectra were collected in the frequency interval 600–800 cm^−1^ (vibrational modes are given by labels in inset); and, (**b**) schematic drafts of the *trans* and *gauche* methionine structures before and after interaction with the Si_3_N_4_ powder with a representation of the main vibrational modes (band labels in inset refer to labels in Fig. 8 and to the vibrational modes given in Table S-I).

In order to confirm the above hypothesis of interaction between charged surface groups on the Si_3_N_4_ surface and the HA methionine terminus of Influenza A H1N1 virions, we performed basic Raman experiments on *L-* and *DL-*methionine in aqueous solution containing 15 wt.% Si_3_N_4_ powder. The Raman spectra of the two above methionine polymorphs before and after interaction with the Si_3_N_4_ particles are given in Fig. 9a for the spectral zone 600 ~ 800 cm^−1^ (cf. labels). Drafts of the HA terminus of the methionine molecule in its *gauche* and *trans* configurations is depicted in Fig. 9b. The labels in the spectra of Fig. 9a, which refer to the deconvoluted virion spectra in Fig. 8, help to visualize important similarities between the spectra of individual methionine polymorphs and that of Influenza A virions, as follows:

(i) A substantial rearrangement of the low-frequency spectral region of the *L-*methionine polymorph containing Bands 3 ~ 5 (cf. upper spectrum in Fig. 9a), with a significant enhancement of Band 4 and the appearance of a new Band 4* in the metastable *DL-*methionine polymorph after treatment with Si_3_N_4_ in aqueous solution (cf. lower spectrum in Fig. 9a).
(ii) A substantial rearrangement of the spectral region containing Bands 7 and 8, which belonged to *DL-* and *L-*methionine, respectively (cf. both Fig. 9a, b), with the appearance of a new Band 8* in the metastable *DL-*methionine polymorph at a frequency corresponding to that observed the Raman spectrum of virions in Fig. 8c.

A likely interpretation of the above spectral features attributes the significant reduction in intensity of Bands 7 and 8 upon methionine interaction with Si_3_N_4_ powder to the formation of thioether groups in the methionine residue, which is thus deprived of the C-S bond on the CH_3_ side to turn into homocysteine (as schematically depicted in Fig. 9b). Such chemical circumstance in turn enhances the vibrational activity of C-S bonds on the CH_2_ side, which can explain the observed enhancement in the intensity of Band 4. This interpretation is supported by the observation of the new Band 20* at around 959 cm^−1^, which corresponds to S–H in-plane bending vibration of homocysteine, and by the increased signal for Bands 18, 19, and 22 also contributed by vibrational modes of homocysteine in the Si_3_N_4_-exposed virions (cf. Fig. 8d and Table S-II). Note also that Band 18 is possibly contributed by the stretching mode of C-COO^−^ deprotonated carboxyl group, which is in line with a pH buffering effect induced by Si_3_N_4_ in the aqueous solution^14^.

**Figure 10:**
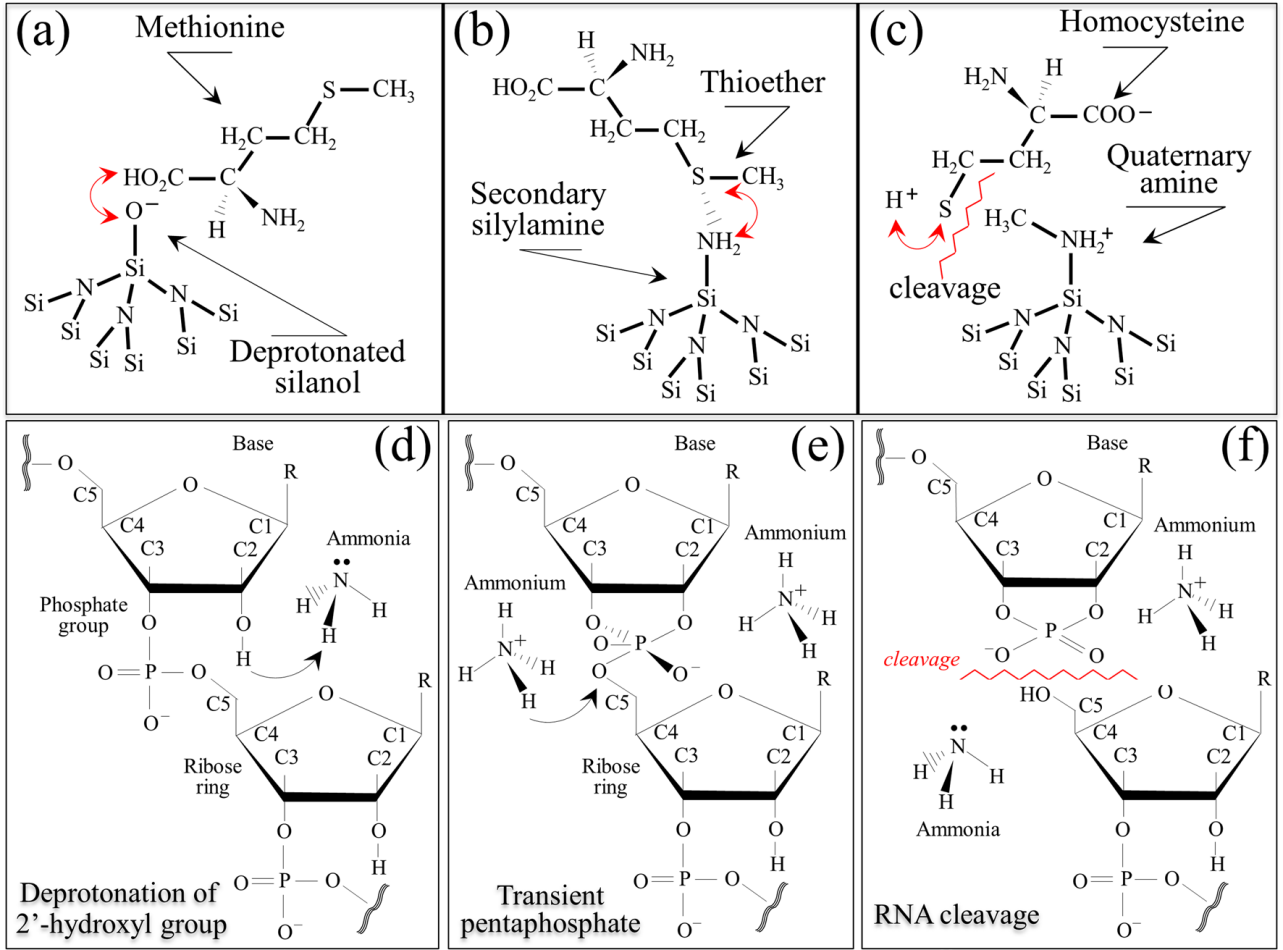
(**a**) Attraction by deprotonated silanol groups at the surface of Si_3_N_4_ toward the C-COOH terminus of methionine; (**b**) formation of hydrogen bond at the thioether with hydrogen bond acceptor silylamine sites on the Si_3_N_4_ surface; and, (**c**) bonding of secondary silylamines to carbon of the methionine methyl group CH_3_ to form a quaternary amine with positive charge, while releasing a homocysteine molecule in the environment. In the lower panels, the process of genome degradation by alkaline transesterification through hydrolysis of RNA phosphodiester bond: (**d**) deprotonation of the 2′-hydroxyl group by ammonia base; (**e**) formation of transient pentaphosphate unit; and, (**f**) interaction with acidic ammonium leading to RNA fragmentation by self-cleavage.

The basic experiments on pure methionine polymorphs in Si_3_N_4_-added aqueous solution suggest that the most probable scenario behind the interaction between Si_3_N_4_ surface and the envelope of Influenza A virions involves deprotonated silanol groups at the surface of Si_3_N_4_ exerting a strong electrostatic attraction toward the C-COOH terminus of the methionine residue (Fig. 10a). On the other side of the methionine molecule, thioether cleavage can be triggered by protonated amino groups, which act as hydrogen bond donors and form strong hydrogen bonds with hydrogen bond acceptor silylamine sites on the Si_3_N_4_ surface (Fig. 10b)^48^. The potential attitude of secondary silylamines to bond to carbon likely involves a direct link with the methionine methyl group CH_3_ to form a quaternary amine with positive charge (Fig. 10b). We thus hypothesize that, once the viral capsid is mechanically forced to detach from the Si_3_N_4_ particle by centrifugation or filtration, the thioether group of methionine residue might cleave while its C-COOH group deprotonates, thus transforming methionine residues into a zwitterionic homocysteine form (Fig. 10c), as detected by Raman spectroscopy.

In addition to S-related bond vibrations, the low-frequency region of the Raman spectrum of virions contains fingerprints of the secondary structure of RNA^49,50^. In this study, bands in this zone exhibited significant changes assignable to conformational perturbations of the ribosylphosphate backbone^51^. For example, the observed reduction in intensity of Band 13 (at ~ 818 cm^−1^), which is related to the O–P–O stretching in RNA backbone^52^ (cf. Fig. 8b, d), points to a trend to phosphodiester cleavage^51^. One possible scenario, which is also supported by RT-PCR analyses showing substantial RNA fragmentation after interaction with Si_3_N_4_ (cf. Table 2), is alkaline transesterification of the RNA backbone through hydrolysis of its phosphodiester bond. In case of alkaline transesterification, the process of genome degradation^53^ starts with the deprotonation of the 2′-hydroxyl group by ammonia base (Fig. 10d), it is followed by the formation of a transient pentaphosphate unit (Fig. 10e) and, upon further interaction with acidic ammonium, ends with RNA fragmentation due to self-cleavage (Fig. 10f). Additional damages to the virion RNA structure could be hypothesized from the complete disappearance of Bands 15, 17, and 21, which belong to ring structures in RNA guanine nucleotide (cf. Fig. 8d), and from the missing Band 2 related to ring vibrations of RNA uracil nucleotide (cf. Fig. 8c).

Finally, note that, unlike the mechanism of methionine degradation (peculiar to Influenza A viruses), the processes of ammonia-induced RNA fragmentation could be common across a number of different ssRNA viruses, independent of their enveloped or non-enveloped structures. Despite significant differences in protein structure among different virions, highly volatile NH_3_ and NO radicals can penetrate the virions and damage their RNA, independent of the capsid structure of the virion^53^. In a previous in situ study on the effect of Si_3_N_4_ hydrolysis on living gram-positive bacteria^13^, Raman spectroscopy similarly revealed damages to the DNA structure by disappearance of Raman band assigned to vibrations in deoxyadenosine triphosphate, ring breathing of guanine, and phosphodiester stretching. This effect was attributed to oxidation of ammonia into hydroxylamine along with the concurrent presence of superoxides and highly volatile radical species such as nitric oxide and peroxynitrite. Additional experiments on different Influenza viral strains of the A and B type are ongoing in order to validate the present interpretation of Raman data.

## Discussion and outlook

### Molecular targets of solid-state viral inactivation

The trimeric surface protein hemagglutinin (HA) plays a critical role in two consecutive steps of viral replication: (i) binding to sialic acid on the cell membrane (as a preliminary step toward endocytosis); and, (ii) inducing membrane fusion through a conformational change under the lowered pH environment of the virion-containing acidified endosome^54^. Some 16 distinct subtypes of HA exist^55^, and the identification of a multivalent antiviral remedy effective against multiple HA subtypes yet represents a challenge. In the context of the above item (i), a compound that mimics sialic acid in HA binding could prove effective in attracting the virus independent of HA subtype. Successful examples of the so-called “competitive binding” approach have been reported in the literature in anti-influenza drug design^56,57^, but reports on solid-state viral inactivators based on this approach are yet hardly found in the published literature. Concerning the above item (ii), alternative approaches to HA inactivation have targeted the fusion function of membrane proteins. For example, tert-butylhydroquinone is a compound that binds to three identical sites on the pre-fusion HA trimer with the effect of stabilizing the proteins, preventing their conformational changes in acidic pH environment, and inhibiting viral/endosome membrane fusion^56^. Targeting drug-binding sites with proteins is a well-known research strategy in virus inactivation^57^. Again, however, viral inactivation mechanisms based on the interactions between the HA trimer and a solid-state compound has so far been an unusual research task.

The RNA polymerase of the Influenza virus consists of a trimer of viral proteins PB1, PB2, and PA it is divided into several domains with well differentiated functions, including polymerase, RNA binding, cap-binding, endonuclease, etc. Its primary function is to synthesize (+) sense viral mRNAs and to replicate the (-) sense viral genome. One additional function consists in capping the synthesized mRNAs with a “snatched” 5′ methyl guanosine cap cleaved and then “stolen” from cellular mRNAs^54^. New compounds, such as marchantin E^58^, have proved capable of inhibiting viral RNA polymerase endonuclease activity, thus preventing proteins from cap snatching and viral RNA from being translated. Direct approaches targeting viral RNA have also been pursued^59^. In this latter context, inactivation of Influenza A viral particles has been achieved upon reaction with artificial ribonucleases^60^. These chemical compounds are capable to directly cleave the viral RNA molecules while causing minimal damage to the structure of surface epitopes. The exploitation of RNA cleavage mechanisms holds general validity against infections caused by a wide variety of RNA viruses.

Si_3_N_4_ was effective as a solid-state virus inactivator, merging the effects of competitive HA binding, thioether cleavage of methionine residues, RNA phosphodiester bond cleavage and nucleotide damage. The presented data suggested that Si_3_N_4_ could provide charged sites on contact, which degrade the structure of HA, and elute NH_4_^+^/NH_3_ molecules that fragment the RNA of virions. However, differences in inactivation kinetics among different ssRNA viruses were detected. Such differences may arise from: (i) different levels of electrical attraction of virions toward the Si_3_N_4_ particles; and, (ii) differences in the genomic structure of different viruses.

Regarding the above item (i), the reported IEP of FCV virus is very close to that of Si_3_N_4_ (cf. Fig. 3). Accordingly, FCV virions possess an electrical charge very close to that of the Si_3_N_4_ surface over a wide range of pH and, thus, they experience a lower probability of encountering a Si_3_N_4_ particle in suspension due to weak electrical attraction. Conversely, Influenza A H1N1 and EV-71 virions possess IEPs significantly greater than that of Si_3_N_4_. Translated in terms of electrical attraction, these latter virions are subjected to attractive forces and experience a higher probability of being “caught” by the Si_3_N_4_ particles. Once in contact with the Si_3_N_4_ surface, the virions are “poisoned” by eluted ammonia molecules. Such a two-steps virion/surface interaction has been found very effective also in the case of SARS-CoV-2 virions, which could be inactivated up to > 99% within exposure times as short as 1 min. This composite antiviral mechanism was branded as the “catch-and-kill” effect^15^.

Regarding possible differences in genomic structures (i.e., the above item (ii)), the heterogeneity observed here among ssRNA viruses toward Si_3_N_4_ inactivation is likely also contributed by differences in sequence and length of the genome^61^. The greater lengths of the FCV genomes (7683 nucleotides^62^) and EV-A71 genomes (7407 nucleotides^63^) compared to that of each genome segment of Influenza A H1N1 (the longest RNA segment consisting of 2341 nucleotides^64^) enhances cleavage probability and may render the former two virions more susceptible to cleavage than the latter one. This factor might partly compensate for a weaker electrical attraction of the FCV toward the Si_3_N_4_ surface. In summary, the EV-A71 virus is most efficiently inactivated because of two concurrent circumstances: a high electrical attraction to the Si_3_N_4_ surface (due to its high IEP) and a high probability of RNA transesterification due to its greater genome length.

**Figure 11:**
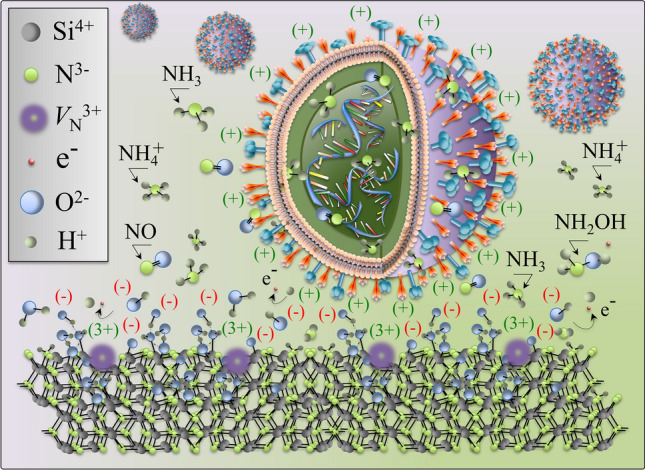
Schematic draft of the dual effect of “catch-and-kill” viral inactivation at the interface between Influenza A virions and Si_3_N_4_: negatively charged silanol groups attract the virions at the HA methionine terminus, while highly volatile NH_3_ (eluted by the Si_3_N_4_ surface) and NO (formed by reactions with ROS from split water) molecules penetrate the virions and damage their RNA sequence by alkaline transesterification and uracil/guanine ring disruption.

A schematic draft of the “catch-and-kill” antiviral effect developed at the interface between Influenza A HIN1 virions and the Si_3_N_4_ particles in aqueous suspension is depicted in Fig. 11. According to this schematic draft, negatively charged silanol groups attract the virions while secondary silylamines “snatch” the C-S bond of the methionine residue at HA terminus. Concurrently, highly volatile NH_3_ molecules (directly eluted by the Si_3_N_4_ surface) swiftly penetrate the envelop of the virions and damage their RNA backbone and nucleotides. This effect has been consistently demonstrated by both in situ Raman spectroscopic assessments and RT-PCR analyses of viral genome.

### Significance of the present findings and future developments

Our previous in vitro studies of bacteria^13^ and mesenchymal cells^65^, as well as retrieval analyses of explanted Si_3_N_4_ spinal inserts^66^ have revealed a dual attitude for Si_3_N_4_; namely, this bioceramic being capable of lysing bacteria while supporting cell proliferation and functionality. This dualism is the result of a cascade of off-stoichiometric reactions that take place at the surface of Si_3_N_4_, and lead to the formation of silanol complexes and elution of NH_4_^+^/NH_3_ species^12,14^. At physiological pH, the main species eluted is NH_4_^+^, which enters the cytoplasmic space of cells in controlled concentrations through specific transporters^67^. Ammonium ions represent a nutrient, which is used by the cells to synthesize building-block proteins for both enzymes and genetic compounds as needed to sustain cell differentiation and proliferation^68^. Conversely, highly volatile ammonia molecules freely penetrate the external membrane and directly target the stability of DNA/RNA structures in both mammalian and bacterial cells^69^. However, at the low concentration and slow kinetics of NH_3_ elution from Si_3_N_4_ at physiological pH, eukaryotic cells receive no damage because their mitochondria can easily neutralize small NH_3_ infiltrations through cytochrome *c* oxidase enzymatic catalysis. This is a reaction chain that produces free electrons and reactive intermediates, which ultimately oxidize NH_3_ into hydroxylamine NH_2_OH (ammonia monooxygenase) and boost NO formation^70^. Conversely, bacteria, which lack mitochondria, cannot catalyze NH_3_ and face chemical destabilization of their DNA/RNA structure^13,14^.

We probed here whether a surface chemistry mechanism similar to DNA/RNA destabilization could be operative versus ssRNA viruses and discovered the two following (combined) antiviral actions for Si_3_N_4_: (i) competitive HA binding; (ii) RNA phosphodiester bond cleavage; and, (iii) RNA nucleotide ring damage (specifically in uracil and guanine). The former mechanism is related to electrostatic attraction exerted by deprotonated surface silanols at the Si_3_N_4_ surface, while both the latter two ones arise from molecular infiltrations of eluted NH_3_. The present study is in line with our previously published data^12–15^, and it specifically confirms our recently published data on the inactivation of SARS-CoV-2 by nitride ceramics^15^. The efficacy of Si_3_N_4_ as a solid-state virus inactivator relies on RNS rather than ROS species^12–14^. Antiviral activities of insoluble solid-state materials have so far been reported for copper, aluminium, and silver compounds^71^. Solid-state cuprous oxide (Cu_2_O) is known to inactivate both enveloped and non-enveloped viruses. Cu_2_O markedly reduces the HA activity by disrupting host cell recognition through denaturing protein structures on viral surfaces, independent of the presence of a viral envelope. However, although solid Cu_2_O shows superior antiviral activity, this compound is quite toxic to cells. A high level of toxicity for copper particles toward VeroE6/TMPRSS2 was also recorded in our recent study on SARS-CoV-2^15^. In aqueous solution, soluble oxygen oxidizes Cu^+^ ions to Cu^2+^ through reactions that produce ROS^72^. ROS then readily react with lipids, proteins, and nucleic acids, and result in significant damage to cell structures by quickly cumulating into a situation of oxidative stress^73^. Cu_2_O crystals were found to promote endothelial cell death via autophagy, to elevate the level of ROS such as superoxides, which subsequently activate AMP-activated protein kinase^74^. Unlike the enhancement of ROS caused by metal contact, cells easily metabolize the RNS developed at the interface with Si_3_N_4_ for a similar if not even superior antiviral effect.

It should be noted that electron microscopy observations of the Si_3_N_4_ particles used in this study (cf. Fig. 1a) revealed that they are composed of many smaller crystallites imparting to the particles a quite rough surface. The dimension of viruses is much smaller than the Si_3_N_4_ particles and comparable with the nanostructures observed on the surface of the particles. This circumstance could provide an additional scavenging effect on the viruses, which likely adds upon the chemical effect and could also be common to other types of ceramic microparticles showing a similar surface structure.

A recent study^75^ revealed high antibacterial efficacy also for Si_3_N_4_ particles embedded in a polymeric matrix despite only a minor fraction of particles was added. Two recent studies on 3D-additive Si_3_N_4_ coating by laser cladding similarly revealed the possibility to translate antipathogenic properties to metallic^76^ and polymeric^77^ surfaces. The results obtained on polymer/ceramic composites and coatings suggest that the antibacterial/antiviral properties of Si_3_N_4_ could directly be translated into virus-resistant surfaces for use in hospitals, schools, and other environments where contact-based viral disease transmission is a concern.

## Conclusion

We described a novel Si_3_N_4_ ceramic technology that was effective in inactivating ssRNA viruses independent of virions being of enveloped or non-enveloped types, or their RNA possessing positive or negative polarity. The favorable surface chemistry of Si_3_N_4_ can control viral transmission without the toxicity associated with other anti-viral strategies. Virus inactivation by Si_3_N_4_ ceramics represents a new, broad-spectrum approach that is safe toward mammalian cells. Its unique combination of surface properties could help control the surface-mediated rapid transmission of viral epidemics. The molecular mechanisms described in our report may be generally effective against other known viruses, and be useful in combatting disease outbreaks.

## Supplementary Information

Supplementary Information
