# Supplementary material for: Silicon nitride: a potent solid-state bioceramic inactivator of ssRNA viruses
Source: Sci Rep. 2021 Feb 3;11:2977. doi: 10.1038/s41598-021-82608-3 (PMC7858580; doi:10.1038/s41598-021-82608-3)
Supplement: Supplementary file 1 — Supplementary Information [file 41598_2021_82608_MOESM1_ESM.docx]

**Silicon nitride: a potent solid-state bioceramic**

**inactivator of ssRNA viruses**

Giuseppe Pezzotti,^a,b,c,d^* Francesco Boschetto,^a,d^ Eriko Ogitani,^d^ Yuki Fujita,^a^ Wenliang Zhu,^a^ Elia Marin,^a,e^ Bryan J. McEntire,^f^ B. Sonny Bal,^f^ and Osam Mazda,^d^

^a^*Ceramic Physics Laboratory, Kyoto Institute of Technology, Sakyo-ku, Matsugasaki, Kyoto 606-8585, Japan;* ^b^*Department of Orthopedic Surgery, Tokyo Medical University, 6-7-1 Nishi-Shinjuku, Shinjuku-ku, 160-0023 Tokyo, Japan;* ^c^*The Center for Advanced Medical Engineering and Informatics, Osaka University, 2-2 Yamadaoka, Suita, Osaka 565-0854, Japan;* ^d^*Department of Immunology, Graduate School of Medical Science, Kyoto Prefectural University of Medicine, Kamigyo-ku, 465 Kajii-cho, Kyoto 602-8566, Japan; ^e^Department of Dental Medicine, Graduate School of Medical Science, Kyoto Prefectural University of Medicine, Kamigyo-ku, Kyoto 602-8566, Japan; ^f^SINTX Technologies Corporation, 1885 West 2100 South, Salt Lake City, UT 84119, USA*

**Supplementary Information**

*1. Characterizations of the antiviral Si_3_N_4_ powder*

Bulk Si_3_N_4_ samples (manufactured by SINTX Corporation; Salt Lake City, UT) were sintered in nitrogen atmosphere at a temperature in excess of 1700^o^C, and densified by hot isostatic pressing under a N_2_ gas pressures >200 MPa and at temperature exceeding 1650^o^C. The produced Si_3_N_4_ mainly consisted of anisotropic β-Si_3_N_4_ grains separated by thin (<2 nm) grain boundaries of amorphous or crystalline yttrium aluminum oxynitride or Si(Y)AlON, respectively. The Si_3_N_4_ powder used in this antiviral study was obtained from the above sintered samples by mechanical grinding the Si_3_N_4_ sintered body and through successive filtration. This grinding/filtration process allowed us to statistically control the particle size to an average diameter of 6 μm with a standard deviation of ± 1.6 μm (cf. Fig. 1(a) of the main manuscript). The observation of micrometric β-Si_3_N_4_ grains with acicular shape gives evidence of their polycrystalline structure.

A photoelectron spectrometer (JPS-9010 MC; JEOL Ltd., Tokyo, Japan) with an X-ray source of monochromatic MgK (output 10 kV, 10 mA) was employed for X-ray photoelectron spectroscopy (XPS) analyses. Prior to characterization, the surface was cleaned by Ar^+^ sputtering in the pre-chamber, while actual measurements were conducted in the vacuum chamber at around 2×10^-7^ Pa with an analyzer pass energy of 10 eV and voltage step size of 0.1 eV. X-ray incidence and takeoff angles were set at 34° and 90°, respectively. Spectra were averaged over ten separate measurements.


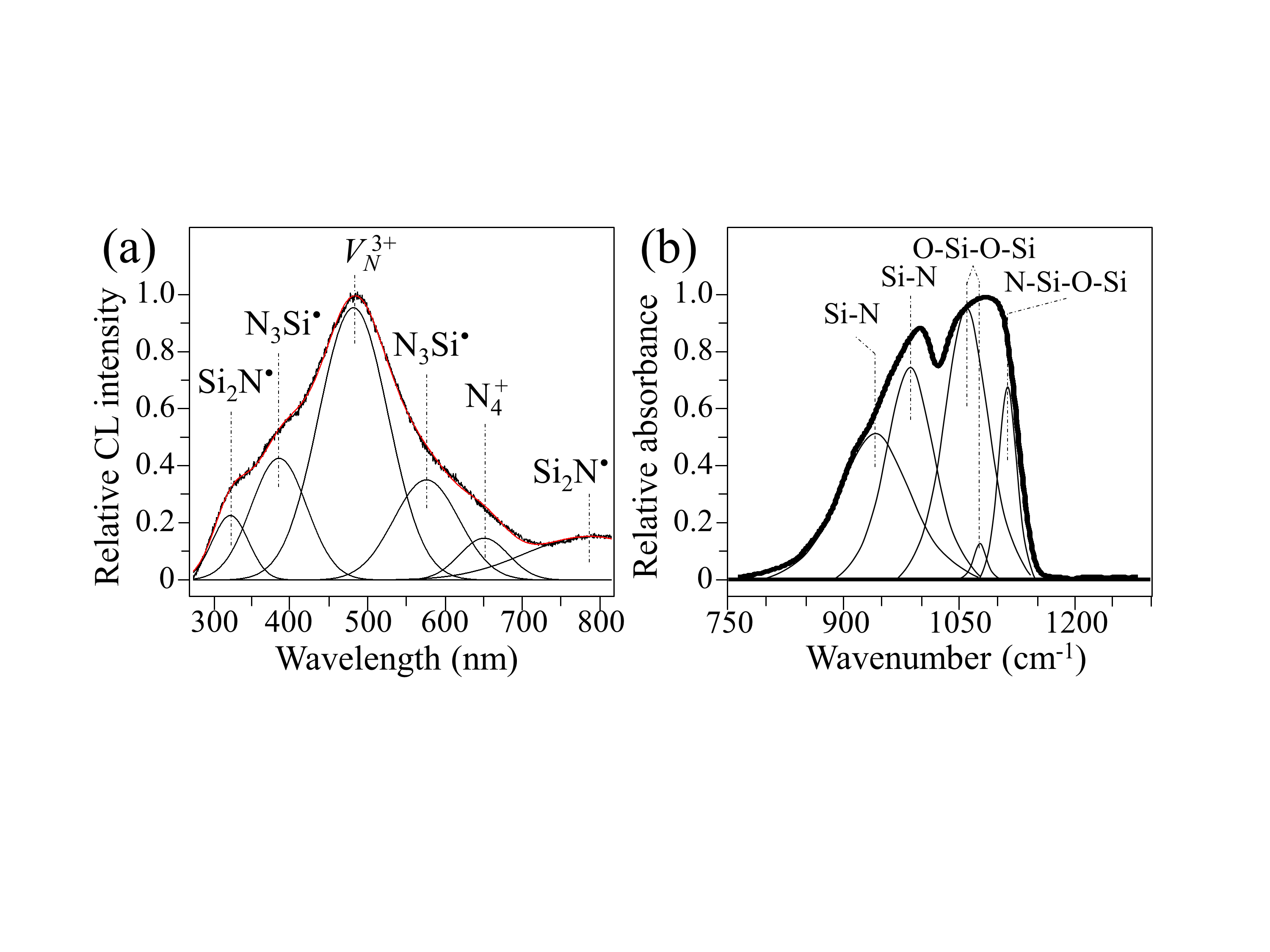


**Fig. S-1:** Characterizations of the Si_3_N_4_ powder: (a) CL spectrum and its deconvolution into 6 sub-bands arising from a variety of off-stoichiometric sites both on N and Si sides (cf. labels); and, (b) ATR-FTIR spectrum deconvoluted into 5 sub-bands that vibrationally characterize the bond population at the Si_3_N_4_ surface (cf. labels).

Cathodoluminescence (CL) spectra were collected using a field-emission gun scanning electron microscope (FEG-SEM, SE-4300, Hitachi Co., Tokyo, Japan) equipped with an optical device. The acceleration voltage and the beam current were fixed at 5 kV and 80 pA, respectively. The CL device consisted of an ellipsoidal mirror and an optical fiber bundle, which served to collect and to address the emitted electron-stimulated luminescence into a highly spectrally resolved monochromator (Triax 320, Jobin-Yvon/Horiba Group, Tokyo, Japan). About 100 CL spectra were randomly obtained from different areas using an acquisition time of 60 s in order to ensure statistical significance. The spectra, which were characteristic of quite shallow portions of material (~5 nm in depth), were deconvoluted using commercial software (Origin 9.1, OriginLab Co., Northampton, MA, USA) and the results averaged and compared. The average CL spectrum is shown in Fig. S-1(a). CL spectroscopy revealed a variety of off-stoichiometric sites both on N and Si sides, which included a main emission from positively charged nitrogen vacancies, *V_N_*^3+^, Si dangling bonds (N_3_Si•), N dangling bonds (Si_2_N•), and N_4_^+^ defects associated with the presence of N-N bonds (cf. labels and Ref. 16 in the main text).

Attenuated total reflection Fourier transform infrared (ATR-FTIR) spectra were also recorded by means of a high sensitivity spectroscope (Spectrum 100FT-IR Spotlight 400; PerkinElmer Inc., Waltham, MA, USA). The spectral resolution of this equipment was 0.4 cm^-1^. Average ATR-FTIR spectra were computed using 6 independent measurements performed on n=3 samples. Spectral acquisition and pre-processing of raw data, which included baseline subtraction, smoothing, normalization and fitting, were carried out using commercially available software (Origin 8.5, OriginLab Co., Northampton, MA, USA). ATR-FTIR data, which vibrationally characterize the bond population at the Si_3_N_4_ surface, are given in Fig. S-1(b). As seen, this additional set of data was consistent with the XPS and CL characterizations and confirmed that the bond population at the surface of β-Si_3_N_4_ sample is comprehensive of two types of bonds: Si-N and Si-O-Si (cf. labels and Ref. [21] in the main text).

*2. Alterations of surface bond population in Si_3_N_4_ powder as a function of pH*

*
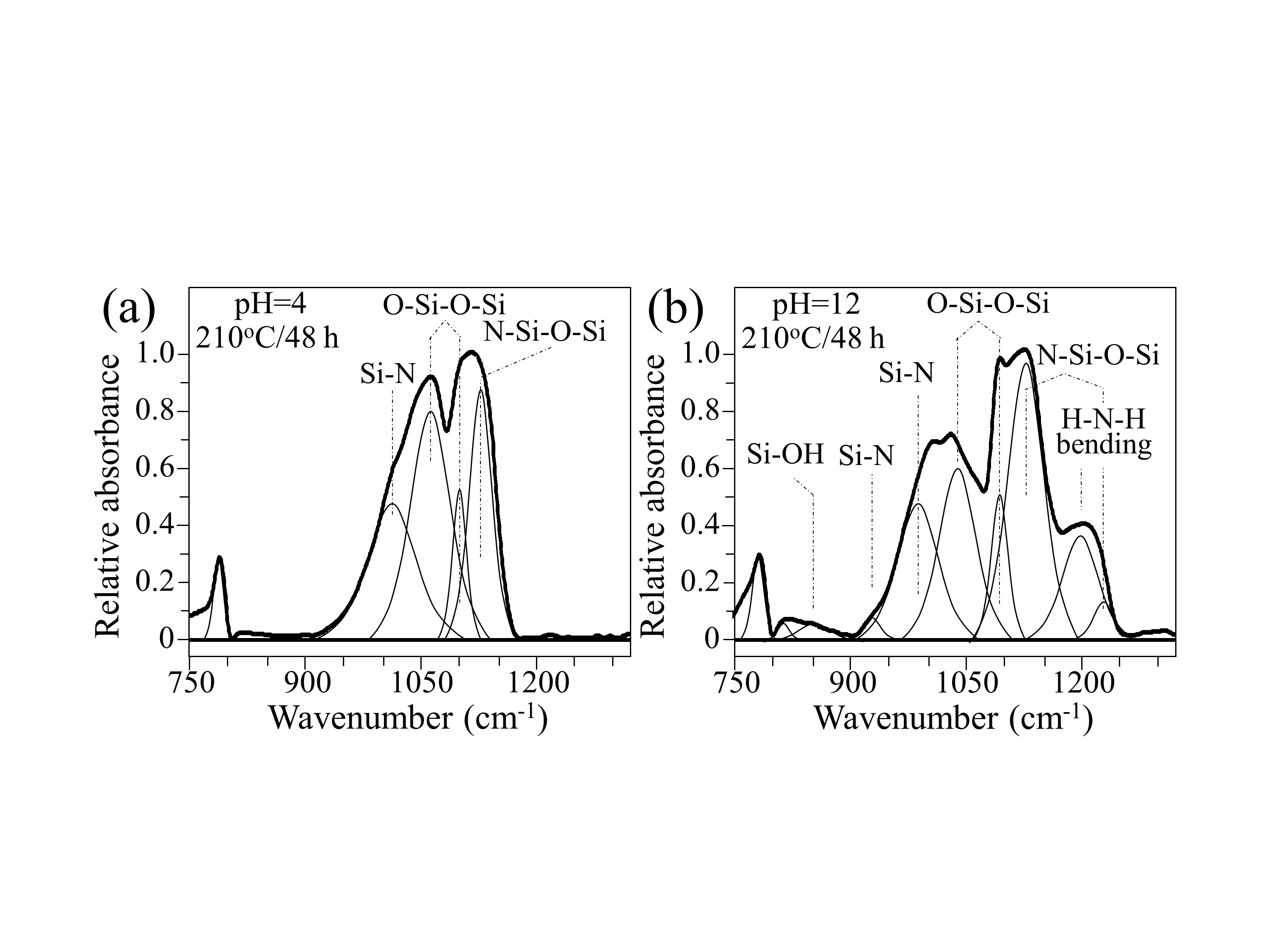
*

**Fig. S-2:** ATR-FTIR deconvoluted spectra of Si_3_N_4_ after 48 h treatment at 210^o^C under environmental pH values of 4 (a) and 9 (b).

Figure S-2 represents ATR-FTIR spectra ((a), and (b)) as collected on powder samples treated for 48 h at 210^o^C under environmental pH values of 4 and 12, respectively (spectra averaged on three samples for each tested condition). The respective spectroscopic characteristics clearly changed according to treatments in different pH. ATR-FTIR deconvoluted spectra, as collected after treatment under acidic environment (in Figs. S-2(a)) revealed that the thermal treatments under different pH did not change the bond nature but only altered the population of the bond structure at the powder surface. Upon treating at pH=4, the altered sub-band balance as well as a shift toward lower wavenumbers pointed at the development of a prevalent population of O-Si-O-Si above N-Si-O-Si bonds (cf. labels in Fig. S-2(a)). Conversely, a more dramatic change in bond population could be observed upon treating under highly alkaline environment (pH 12; Fig. S-2(b)). Contrary to the case of pH 4, the total absorbance by a population of N-Si-O-Si increased, but also a clear increase in absorbance for the high-frequency O-Si-O-Si band could be observed. The trend of this band, which represents asymmetric stretching mode, can be attributed to an induced reordering at the surface toward increased SiN_4_ bonding. Moreover, the alkaline treatment introduced new types of bonds, which included a Si-OH low-frequency absorbance at ~882 cm^-1^ and a H-N-H bending mode with absorbance at 1230 cm^-1^ (cf. labels) [S-1].

From a surface charge viewpoint, the surface modifications as a function of pH treatment modified the bond population, but did not significantly alter the IEP of Si_3_N_4_. Zeta potential measurements were performed using an electrokinetic analyzer (SurPASS, Anton-Paar USA, Ashland, VA). A background electrolyte of 1 mM HCl, which exhibited a natural pH of 5.5, was used in all experiments. Experiments were divided into two runs. The first run took measurements across a pH range of 5.5–3 using auto-titration of 0.1 M HCl solution to control pH. The second run used a new solution of background electrolyte for measurements across a pH range of 5.5-10 and auto-titration of 0.1 M NaOH solution to control pH. Each run contained two material samples. Observed streaming potentials were converted into zeta potentials using the Helmholz-Smoluchowski equation. The results of IEP measurements on the present Si_3_N_4_ material are given in Fig. 3 of the main text. None of the variations in bond population induced by the present thermal treatments significantly affected the IEP (cf. main text). The present thermal treatments of the Si_3_N_4_ powder in acidic and alkaline environments partially altered the surface bond population but did not significantly change the density of deprotonated SiO^-^ sites and thus the IEP of the surface. As a result, the net surface charge remained negative in the neighborhood of homeostatic pH, with a minor fraction of positively charged sites stemming from both SiNH_3_^+^ species and 3+ charged N-vacancies. A comparison between the IEPs of investigated Si_3_N_4_ powder and those of the selected viral strains is given in Fig. 3 of the main text.

*3. Cells, viruses, and immunochemistry characterizations*

Cells and viruses

MDCK cells (Madin-Darby canine kidney cell) were purchased from DS Pharma Biomedical Co., Ltd. (Suita, Japan). The MDCK cells were cultured in DMEM (Nacalai Tesque, Kyoto, Japan) supplemented with 4% FBS, 100 U/ml penicillin, and 100 μg/ml streptomycin (Complete Medium) and plated in 6-well plate at 6×10^5^ cells/well for plaque assay, or loaded on glass based dishes (TECHNO GLASS Co., Shizuoka, Japan) at 1×10^6^ cells/dish for immunochemistry assays. Cells were cultured in Complete Medium at 37^o^C in an atmosphere containing 5% CO_2_.

The CRFK (feline kidney) cell line was purchased from Health Science Research Resources Bank (Sennan, Japan), while the Rhesus monkey kidney (LLC-MK2) cell line was purchased from ATCC. Both types of cell were cultured in DMEM supplemented with 10% FBS, 100 U/ml penicillin, and 100 μg/ml streptomycin and plated in 96-well plate at 3×10^4^ cells/well for TCID_50_ assay.

The influenza A virus, A/Puerto Rico/8/34(H1N1) (PR8) strain, was obtained from the Virus Research Center, National Institute of Infectious Diseases. The HEV71 (ATCC VR­1432) and the FCV F-9 strain (ATCC VR-782) were directly purchased from ATCC. A brief description of the viral strains used in this study is given in the following.

*Influenza A H1N1*

Influenza A H1N1 virus is an enveloped virus that belongs to the *Orthomyxoviridae* family. It contains negative-stranded RNA genomes with eight RNA segments encoding ten viral proteins. The three largest segments encode the polymerase acidic protein and the polymerase basic proteins 1 and 2. These three proteins assemble to form a heterotrimeric viral RNA-dependent RNA polymerase with transcription and replication functions. In contrast, viral hemagglutinin (HA) and neuraminidase (NA) are the essential envelope proteins responsible for viral entry into, and budding from, cells, respectively.

The IEP of HA in the Influenza A virus has been measured as 7.47 [S-2]. Accordingly, the trimeric viral HA protein charges positive at physiological (and lower) pH and promptly binds to the negatively charged sialic acid in adhering to host cells. Influenza A can evolve and recombine into new antigenic forms, allowing it to evade immunity associated with vaccination or with previous natural infections. Once spread into the human population, influenza viruses can quickly undergo mutations, resulting in antigenic drift. Worldwide pandemics can follow, such as the 2009 H1N1 swine flu epidemic [S-3, S-4].

Influenza antigenic drifts and shifts present a moving target to immune systems, and with zoonoses from animal reservoirs, the virus constitutes a well-known epidemiological risk. Accordingly, intensive and continuous surveillance of influenza viruses is carried out in non-human hosts. The most recent examples of such monitoring revealed resistance to M2 ion channel blockers amantadine and rimantadine, and resistance to the neuraminidase inhibitor oseltamivir in avian Influenza A viruses [S-5, S-6].

*Feline calicivirus*

The FCV is a non-enveloped and positive-sense ssRNA virus belonging to the family *Caliciviridae*. This virus induces infection in the upper respiratory tract of cats and can lead to ulcerative stomatitis. Its open reading frames (ORFs) encode a helicase, a protease, and polymerase (ORF 1), a capsid protein (ORF 2), and an RNA-associated structural protein (ORF 3). Although the FCV capsid protein is the target of protective immune responses, hypervariable regions in this gene confer antigenic variability and, thus, viral persistence. Accordingly, FCV has the capacity of prompt antigenic drifts [S-7].

While not a human pathogen, FCV has a strong similarity to the Norovirus, a highly contagious virus that causes human disease. Unlike Norovirus, FCV can be infected and amplified in cultured cells *in vitro*, making it suitable for research targeted at understanding Norovirus. Unlike the Influenza A virion, the surface of FCV charges strongly negative at physiological pH, thus FCV surface IEP can be as low as 3.9 [S-8]. The existence of FCV heterologous strains hinders a full protection through vaccination [S-9, S-10], and yet the antigenic distance between the vaccine and challenge strains is large.

*Enterovirus 71*

The human EV-A71, a member of the *Picornaviridae* family, is a positive stranded non-enveloped virus with a typically icosahedral morphology. This virus has only recently evolved with the first known strain being isolated in 1965. It is represented by strains falling into six genogroups named A to F [S-11]. Three genogroups (A, B, C), including different subgenogroups (B0–B5, C1–C5), have been defined on the basis of viral protein 1 sequences, while additional genogroups (D, E, F, G) have later been proposed [S-12-S-13].

For the present experiments, we purchased the HEV 71 (species Enterovirus A, genus Enterovirus, family Picornaviridae) viral strain directly from the ATCC (EV-A71 VR-1432 “Vesicular fluid from an adult female with hand, foot, and mouth disease, Wuhan, China), which is a common etiologic agent of hand, foot and mouth disease in pediatric patients [S-14]. Similar to other *picornaviruses*, EV-A71 is a positive ssRNA virus whose genomic sequence is made up of 7411 base pairs and has an open reading frame flanked on both ends by the 5’ Non-Translated Region and the 3’ Non-Translated Region [S-15, S-16].

Amino acid substitutions at different positions in the EV-A71 genome can arise spontaneously, and confer virulence of different subgenotype strains [S-17]. Although viruses with very alkaline IEP are quite rare [S-18], the surface of EV-A71 possesses an IEP as high as 10.9 [S-19]. Accordingly, it charges strongly positive at physiological pH. EV-A71 viral infections can lead to encephalitis, meningitis, and poliomyelitis-like paralysis [S-20]. Recent epidemics in the Asia-Pacific region have manifested with a severe form of brainstem encephalitis and associated with three genetic lineages undergoing rapid evolutions. More details about the evolutionary changes of this virus have been reported in a review paper by McMinn [S-21].

Molecular surveillance of enteroviruses can help to identify new variant strains with increased virulence and pathogenicity [S-22]. While no antiviral agent is effective against EV-A71, both bovine and human lactoferrins can inhibit its infection [S-23].

Si_3_N_4_ powder exposure-test of viruses

The Si_3_N_4_ powder was added to the virus solution to a concentration of 15 wt.% or 30 wt.%, followed by mixing for 1, 5, 10, and 30 minutes at room temperature (RT) or 4°C using a rotating equipment. After centrifugation at 12,000 rpm for 2 minutes at 4°C, the viral infectivity of the supernatant was compared with that of sham exposure by plaque assay (Influenza A H1N1) or TCID50 assay (FCV and HEV71). A schematic draft of the inoculation and subsequent testing/characterization procedures is offered in Fig. S-3.


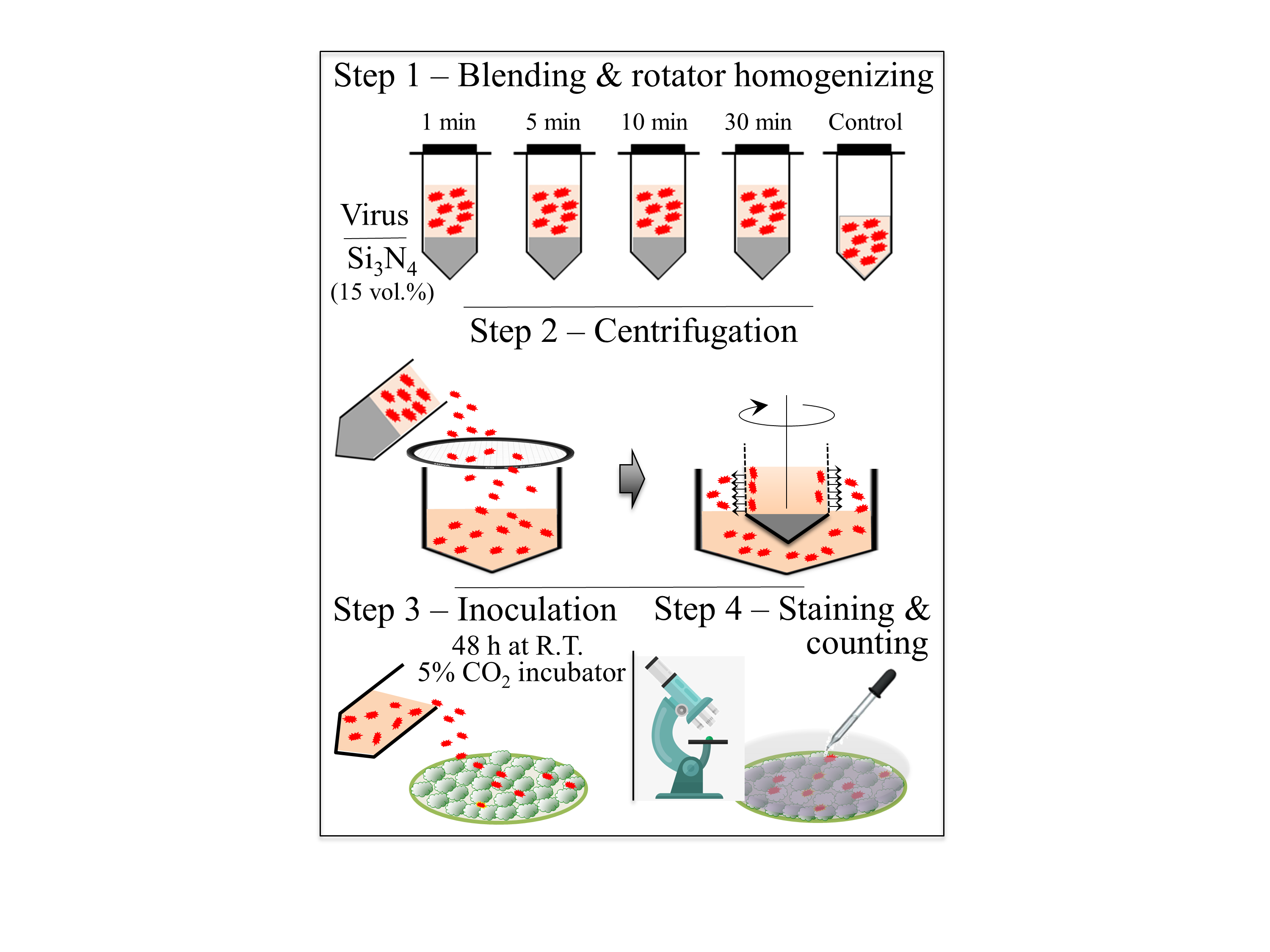


**Fig. S-3:** Procedure of virus inactivation, inoculation and fluorescence microscopy analyses.

Plaque assay

A conﬂuent monolayer of MDCK cells in a 6-well plate was washed twice with serum-free DMEM (SF DMEM), followed by infection with 100 μl of virus suspension in a tenfold serial dilution. After incubation at 37^o^C for 1 h with tilting every 10 min, unabsorbed inoculum was removed, and infected cells were overlaid with 4 ml of DMEM containing 2.5 μg/ml trypsin (Sigma-Aldrich Co. LLC, Saint Louis, USA) and 0.2% albumin (Wako Pure Chemical Industries, Ltd., Osaka, Japan). The plate was incubated at 37^o^C in an atmosphere of 5 % CO_2_ for 2 days. For plaque counting, cells were ﬁxed with 5% glutaraldehyde solution for 2 h, the agarose medium was removed, and the cells were stained with 1% crystal violet.

TCID_50_ assay

After removal of the culture supernatant, a conﬂuent monolayer of CRFK or LLC-MK2 cells in a 96-well plate was inoculated with 50 μl of virus suspension in a tenfold serial dilution. After incubation at 37°C for 1 h with tilting every 10 min, unabsorbed inoculum was removed, and infected cells were added with 100 μl of DMEM containing 4% FBS, 0.2% albumin and 2.5 μg/ml of trypsin. The plate was incubated at 37°C in an atmosphere of 5% CO_2_ for 6-8 days. Cytopathic effect (CPE) was observed in all wells, and TCID_50_ calculated by the Reed-Muench method.

Statistical analyses of the results were carried out by means of the unpaired Student’s *t*-test. The statistical significance of the data was evaluated as highly statistically relevant (*p* < 0.01; labeled with two asterisks), statistically relevant (*p* < 0.01; labeled with one asterisk), or non-significant (*n.s.*).

Fluorescence microscopy

In order to confirm the infectivity of viruses exposed or unexposed to Si_3_N_4_ particles, we visualized MDCK cells inoculated with Influenza A virions exposed and non-exposed to Si_3_N_4_ powder in the fluorescence microscope after washing the infected cells with TBS (20 mM Tris-HCl pH 7.5, 150 mM NaCl), fixing them with 4% paraformaldehyde for 10 min at RT, and permeabilizing them with 0.1% triton X in TBS for 5 minutes at RT. Successively, the cells were blocked with 2% skim milk in TBS for 60 min at RT, and stained with mouse anti-influenza A virus nucleoprotein antibody (red) (AA5H ab20343, Abcam, Cambridge, UK) (Dilution =1:500) and fluorescence signals from cell F-actin (green) (Thermo Fisher Scientific) (Dilution =1:500) for 60 min at RT. After washing with a washing buffer, cells were incubated with an Alexa 549 Goat Anti-mouse IgG F(ab')2 (Thermo Fisher Scientific, MA, USA) (Dilution =1:250) for 60 min at RT in the dark. Three samples each were prepared for Si_3_N_4_ exposed and non-exposed tests, respectively. Three visual fields were observed of each sample under a fluorescence microscope (BZX710; Keyence, Osaka, Japan), subsequently the total number of cells and the number of infected cells were calculated using Keyence BZ-X Analyzer. The full set of fluorescence images on cell cultures inoculated with untreated and Si_3_N_4_-treated Influenza A H1N1 virions are shown in Figs. S-4 and S-5, respectively.


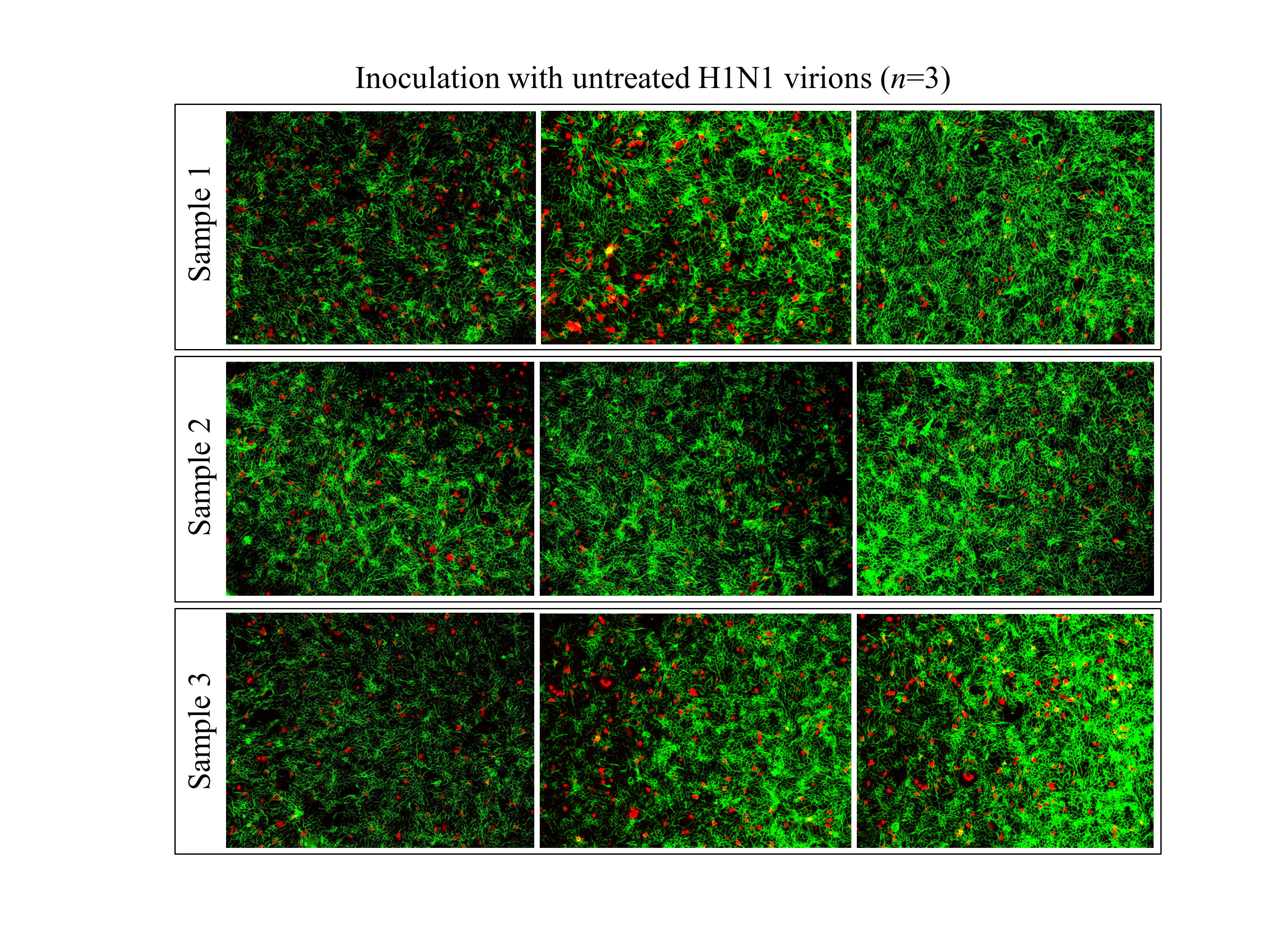


**Fig. S-4:** Full set of fluorescence images of MDCK cells inoculated with Influenza A virus H1N1 virions (control sample); mouse anti-influenza A virus nucleoprotein antibody in red and cell F-actin in green.


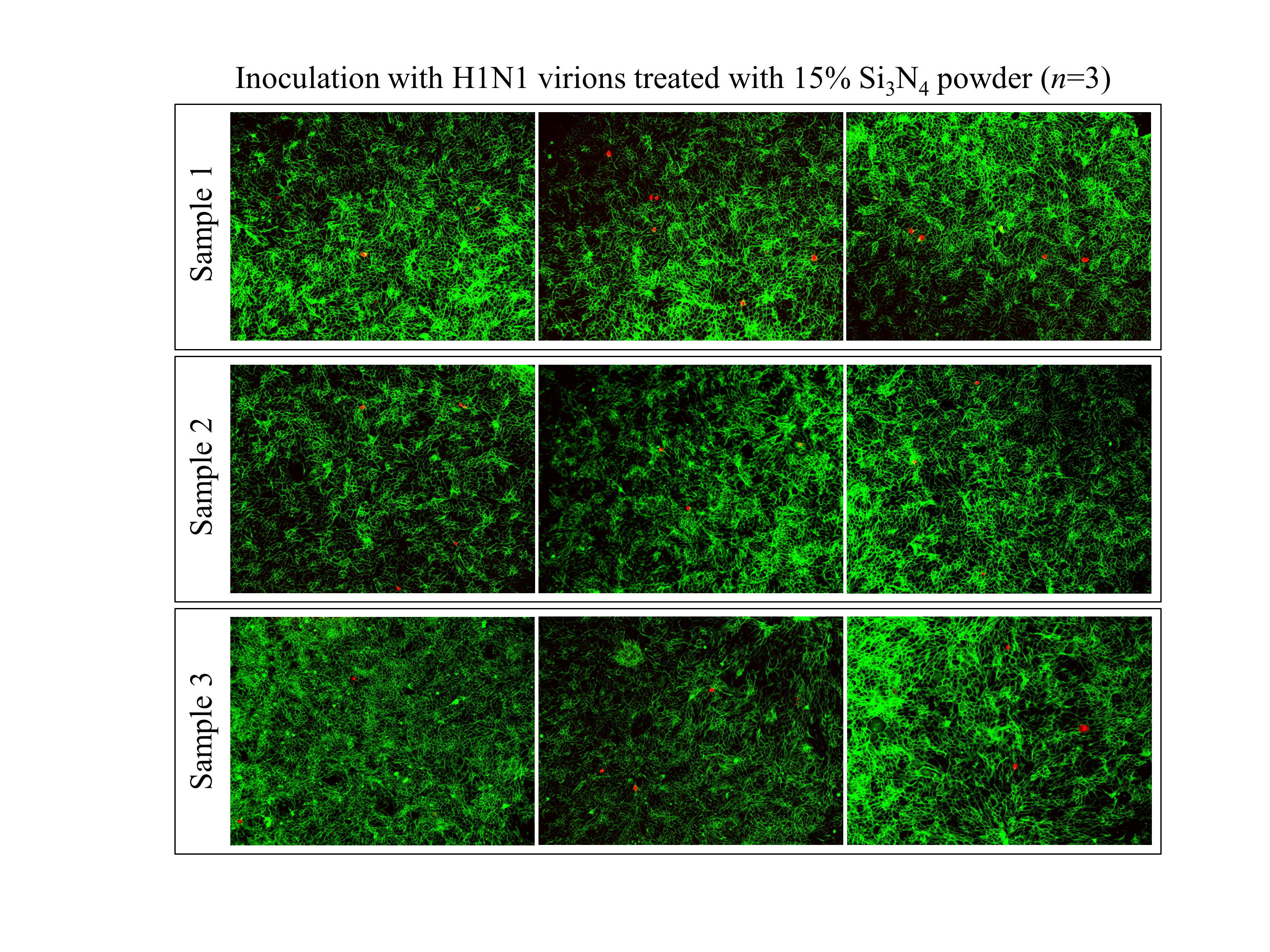


**Fig. S-5:** Full set of fluorescence images of MDCK cells inoculated with Influenza A virus H1N1 virions pre-treated for 10 min in aqueous solution containing a dispersion of 15 wt.% Si_3_N_4_ particles; mouse anti-influenza A virus nucleoprotein antibody in red and cell F-actin in green.

Statistical analyses of the results were carried out by means of the unpaired Student’s *t*-test (*p* < 0.001; labeled with three asterisks).

*4. Real time quantitative RT-PCR test*

The RNA of viruses exposed or unexposed to Si_3_N_4_ particles was extracted using a QI Amp Viral RNA Mini Kit (QIAGEN N.V., Hilden, Germany) according to the manufacturer protocol*.* Reverse transcription was performed using a **ReverTra Ace qPCR RT Master Mix (**TOYOBO CO., LTD.**, Osaka, Japan). 8** μl of RNA template was added to 2 μl of 5 X RT Master Mix. The mixture was incubated at 37 °C for 15 min, 50^o^C for 5 min, 98°C for 5 min and 4°C hold.

We selected primers and probes for Influenza A H1N1 virus based on genomic regions highly conserved in various subtypes and genotypes of the Influenza virus A (matrix protein gene). The selection of the primers and probes was made based on Ref. [57] of the main text. The forward and reverse primers (INFA-1, INFA-2, and INFA-3) are shown in Table I of the main text. The fluorogenic probe for the Influenza A virus consisted of oligonucleotides with the 5’ reporter dye 6-carboxyfluorescein (FAM) and the 3’ quencher dye 6-carboxytetramethylrhodamine (TAMRA). A 20-μl PCR was performed using 2 μl of cDNA, 10 μl of KAPA PROBE FAST qPCR Master Mix (KAPA BIOSYSTEMS, Massachusetts, USA) containing ROX, 900 nM each influenza virus A primer, and 100 nM each probe. Amplification and detection were performed with an ABI StepOnePlus Real Time PCR System (under the following conditions: 1 cycle of 20 sec at 95°C, 45 cycles of 1 sec at 95°C and 20 sec at 60°C. During amplification, the System detector monitored real-time PCR amplification by quantitatively analyzing fluorescence emissions. The reporter dye (FAM) signal was measured against the internal reference dye (ROX) signal to normalize for non-PCR-related fluorescence fluctuations occurring from well to well. The threshold cycle represented the refraction cycle number at which a positive amplification reaction was measured and was set at 10 times the standard deviation of the mean baseline emission calculated for PCR cycles 3 to 15. The quantitative results of Real time quantitative RT-PCR test on Influenza A H1N1 virus are given in Table II of the main text.

*5. Raman spectroscopy of viral strains*

*In situ* Raman spectra were collected using a highly sensitive instrument (LabRAM HR800, Horiba/Jobin-Yvon, Kyoto, Japan) with a 20× optical lens. The spectroscope operated in microscopic measurement mode with confocal imaging in two dimensions. A holographic notch filter within the optical circuit was used to efficiently achieve high-resolution spectral acquisitions. A spectral resolution of 1.5 cm^-1^ was obtained using a 532 nm excitation source operating at 10 mW. The Raman emission was monitored by means of a single monochromator connected to an air-cooled charge-coupled device (CCD) detector (Andor DV420-OE322; 1024 × 256 pixel). The acquisition time was fixed at 10 s. Thirty spectra were collected and averaged at each analysis time-point. Raman spectra were deconvoluted into Gaussian–Lorentzian sub-bands using commercially available software (LabSpec 4.02, Horiba/Jobin-Yvon, Kyoto, Japan). Table S-I gives the exact vibrational frequencies and physical origins of all the labeled bands in the spectra of Figs. 8(a)~(d) (cf. references listed below the Supplementary Tables S-I and S-II).

**Table S-I:** Vibrational frequencies, physical origins and references for all deconvoluted Raman bands labeled in Figs. 8(a) and (c).

| **Band** | **cm^-1^** | **Physical origin** | **Ref.** |
| --- | --- | --- | --- |
| 1 | 604 | C=O bending in RNA cytosine & guanine | (1) |
| 2 | 621 | Ring deformation in RNA uracil | (1) |
| 3 | 640 | C-C-S stretching in methionine (CH_2_ side; *gauche*) | (2) |
| 4 | 650 | C-S stretching in methionine (CH_2_ side; *gauche*) | (2) |
| 4* | 662 | C-C-S stretching in methionine (CH_2_ side; thioether conf.) | (3) |
| 5 | 669 | C-S stretching in methionine (CH_2_ side; *trans*) | (2) |
| 6 | 687 | Pyrrole ring deformation in RNA adenine & guanine | (1) |
| 7 | 698 | C-S stretching in methionine (CH_3_ side; *gauche*) | (2) |
| 8 | 716 | C-S-C stretching in methionine (CH_3_ side; *trans*) | (2) |
| 8* | 728 | C-S-C stretching in methionine (CH_3_ side; thioether conf.) | (3) |
| 9 | 748 | Benzene ring breathing in RNA adenine  C_β_H_2_ rocking in methionine | (1)  (2) |
| 10 | 756 | Ring deformation in RNA cytosine | (1) |
| 11 | 772 | C_β_H_2_ rocking in methionine | (2) |
| 12 | 792 | Ring breathing in RNA cytosine  C_α_H_2_ rocking in methionine | (1)  (2) |

(1) Madzharova F, Heiner Z, Gühlke M, and Kneipp J. Surface-enhanced hyper-Raman spectra of adenine, guanine, cytosine, thymine, and uracil. J. Phys. Chem. C 120: 15415-15423 (2016).

(2) Zhu G, Zhu X, Fan Q, Wan X. Raman spectra of amino acids and their aqueous

solutions. Spectrochim. Acta Part A 78: 1187-1195 (2011).

(3) Gunasekaran S, Bright A, Renuga Devi TS, Arunbalaji R, Anand G, Dhanalakshmi J, Kumaresan S. Experimental and semi-empirical computations of the vibrational

spectra of methionine, homocysteine and cysteine. Arch. Phys. Res. 1: 12-26 (2010).

**Table S-II:** Vibrational frequencies, physical origins and references for all deconvoluted Raman bands labeled in Figs. 8(b) and (d).

| **Band** | **cm^-1^** | **Physical origin** | **Ref.** |
| --- | --- | --- | --- |
| 13 | 818 | O-P-O stretching in RNA backbone (gauche configuration) | (1) |
| 14 | 847 | Ring deformation in RNA tyrosine | (2) |
| 15 | 872 | 5-ring & 6-ring deformation in RNA guanine | (2) |
| 16 | 881 | C-C stretching & CH_2_ rocking in methionine | (3) |
| 17 | 889 | Ring deformation in RNA guanine | (2) |
| 18 | 913 | 6-ring deformation in RNA adenine  S-H in-plane bending in homocysteine  C-COO^-^ stretching in deprotonated carboxyl group | (2)  (4)  (5) |
| 19 | 929 | Ring deformation in RNA adenine  C-C stretching in homocysteine | (2)  (4) |
| 20 | 940 | C-N stretching in amino acids | (3) |
| 20* | 959 | S-H in-plane bending in homocysteine  O-P-O symmetric stretching in adenosine monophosphate | (4)  (6) |
| 21 | 970 | 5-ring deformation in RNA guanine | (2) |
| 22 | 985 | Amide III  S-H in-plane bending in homocysteine | (1)  (4) |
| 23 | 1004 | Ring stretching in RNA guanine  C-SH in-plane bending in homocysteine | (2)  (4) |

(1) Thomas Jr. GJ. Raman spectroscopy and virus research. Appl. Spectrosc. 30: 483-494 (1976).

(2) Madzharova F, Heiner Z, Gühlke M, and Kneipp J. Surface-enhanced hyper-Raman spectra of adenine, guanine, cytosine, thymine, and uracil. J. Phys. Chem. C 120: 15415-15423 (2016).

(3) Zhu G, Zhu X, Fan Q, Wan X. Raman spectra of amino acids and their aqueous

solutions. Spectrochim. Acta Part A 78: 1187-1195 (2011).

(4) Gunasekaran S, Bright A, Renuga Devi TS, Arunbalaji R, Anand G, Dhanalakshmi J, Kumaresan S. Experimental and semi-empirical computations of the vibrational spectra of methionine, homocysteine and cysteine. Arch. Phys. Res. 1: 12-26 (2010).

(5) E. Podstawka, Y. Ozaki, and L.M. Proniewicz, Part II: Surface-enhanced Raman

spectroscopy investigation of methionine containing heterodipeptides adsorbed on

colloid silver, Appl. Spectrosc. 58, 581-590 (2004).

(6) Rimai L, Cole T, Parsons JL, Hickmott JT, Carew EB. Studies of Raman spectra of water solutions of adenosine tri‐, di‐, and monophosphate and some related compounds. Biophys. J. 9: 320-329 (1969).

**References:**

[S-1] V.N. Antsiferov, V.G. Gilev, and V.I. Karmanov, Infrared spectra and structure of Si_3_N_4_, Si_2_ON_2_, and sialons, *Refractories & Ind. Ceram.* **44,** 108-114 (2003).

[S-2] Influenza Research Database (file EF541421)

<https://www.fludb.org/brc/fluSegmentDetails.spg?ncbiGenomicAccession=EF541421&decorator=influenza>

[S-3] W.S. Ryu, *Molecular Virology of Human Pathogenic Viruses* (Elsevier Science, Amsterdam, The Netherlands, 2016) pp. 195-210.

[S-4] G.J. Smith, D. Vijaykrishna, J. Bahl, S.J. Lycett, M. Worobey, O.G. Pybus, S.K. Ma, C.L. Cheung, J. Raghwani, S. Bhatt, et al., Origins and evolutionary genomics of the 2009 swine-origin H1N1 influenza A epidemic, *Nature* 459, 1122-1125 (2009).

[S-5] L.V. Gubareva, L. Kaiser, F.G. Hayden, Influenza virus neuraminidase inhibitors, *Lancet* **355,** 827-835 (2000).

[S-6] G.G. Hayden, Amantadine and rimantadine: clinical aspects. In: *Antiviral drug resistance*. Edited by D.D. Richman (New York: Wiley, 1996) pp. 59-77.

[S-7] C. Baulch-Brown, D.N. Love, J. Meanger, Sequence variation within the capsid protein of Australian isolates of feline calicivirus, *Vet. Microbiol.* **68,** 107-117 (1999).

[S-8] I. Samandoulgou, I. Fliss, J. Jean, Zeta potential and aggregation of virus-like particle of human Norovirus and feline calicivirus under different physicochemical conditions, *Food Environ. Virol.* **7,** 249-260 (2015).

[S-9] A. Lauritzen, O. Jarrett, M. Sabara, Serological analysis of feline calicivirus isolates from the United States and United-Kingdom, *Vet. Microbiol.* **56,** 55-63 (1997).

[S-10] N.C. Pedersen, J.B. Elliott, A. Glasgow, A. Poland, K. Keel, An isolated epizootic of hemorrhagic-like fever in cats caused by a novel and highly virulent strain of feline calicivirus. *Vet. Microbiol.* 73, 281-300 (2000).

[S-11] M. Bessaud, R. Razafindratsimandresy, A. Nougairede, M.-L. Joffret, J.M. Deshpande, A. Dubot-Peres, J.M. Heraud, X. de Lamballerie, F. Delpeyroux, and J.L. Bailly, Molecular comparison and evolutionary analyses of VP1 nucleotide sequences of new African human enterovirus 71 isolates reveal a wide genetic diversity, *PLoS ONE*. **9**, e90624 (2014).

[S-12] B.A. Brown, M.S. Oberste, J.P. Alexander, Jr., M.L. Kennett, M.A. Pallansch, Molecular epidemiology and evolution of enterovirus 71 strains isolated from 1970 to 1998, *J Virol.* **73,** 9969-9975 (1999).

[S-13] V.K. Saxena, S. Sane, S.S. Nadkarni, D.K. Sharma, J.M. Deshpande, Genetic diversity of enterovirus A71, India, *Emerg. Infect Dis.* **21,**123-126 (2015).

[S-14] T. Solomon, P. Lewthwaite, D. Perera, M. J. Cardosa, P. McMinn, M. H. Ooi, Virology, epidemiology, pathogenesis, and control of enterovirus 71, *Lancet Infect Dis.* **10,** 778-790 (2010).

[S-15] R. Andino, N. Böddeker, D. Silvera, A.V. Gamarnik, Intracellular determinants of picornavirus replication,  *Trends Microbiol*. **7,** 76–82 (1999).

[S-16] B.A. Brown, M.A. Pallansch, Complete nucleotide sequence of enterovirus 71 is distinct from poliovirus,  *Virus Res.* **39,** 195-205 (1995).

[S-17] Y. Liu, C. Fu, S. Wu, C. Xiong, Y. Shi, B. Zhou, L. Zhang, F. Zhang, Z. Wang, Y. Zhang, C. Fan, S. Han, J. Yin, B. Peng, W. Liu, X. He, A novel finding for enterovirus virulence from the capsid protein VP1 of EV71 circulating in mainland China, *Virus Genes* **48,** 260-272 (2014).

[S-18] B. Michen and T. Graule, Isoelectric points of viruses, *J. Appl. Microbiol.* **109,** 388-397 (2010).

[S-19] C.H. Schein, M. Ye, A.V. Paul, M. S. Oberste, N. Chapman, G.J. van der Heden van Noort, D.V. Filippov, K.H. Choi, Sequence specificity for uridylylation of the viral peptide linked to the genome (VPg) of enteroviruses, *Virol.* **484,** 80-85 (2015).

[S-20] J.L. Melnick, Enterovirus type 71 infections: a varied clinical pattern sometimes mimicking paralytic poliomyelitis, *Rev. Infect. Dis.* **6 Suppl. 2,** S387-S390 (1984).

[S-21] P. McMinn, An overview of the evolution of enterovirus 71 and its clinical and public health significance, *FEMS Microbiol. Rev.* **26,** 91-107 (2002).

[S-22] S. Boettcher, P.E. Obermeier, K. Neubauer, S. Diedrich, Recombinant Enterovirus A71 subgenogroup C1 strains, Germany, 2015, *Emerging Inf. Dis.* **22,** 1843-1846 (2016).

[S-23] T.Y. Lin, C. Chu, C.H. Chiu, Lactoferrin inhibits Enterovirus 71 infection of human embryonal rhabdomyosarcoma cells *in vitro*, *J. Infect. Dis*. **186**, 1161-1164 (2002).
